# The Nucleolus in Human Disease: Ribosome Biogenesis, Nucleolar Surveillance, and Therapeutic Opportunities

**DOI:** 10.3390/biom16081121

**Published:** 2026-07-31

**Authors:** Olivia Delfino, Jiachen Xuan, Nadine Hein, Rita Ferreira, Ross D. Hannan, Amee J. George

**Affiliations:** 1 Genome Sciences and Cancer Division, The John Curtin School of Medical Research, The Australian National University, Australian Capital Territory, Canberra, ACT 2601, Australia; olivia.delfino@anu.edu.au (O.D.); jiachen.xuan@anu.edu.au (J.X.); nadine.hein@anu.edu.au (N.H.); rita.ferreira@anu.edu.au (R.F.); ross.hannan@anu.edu.au (R.D.H.); 2 Menzies Institute for Medical Research, University of Tasmania, Hobart, TAS 7000, Australia

**Keywords:** nucleolus, ribosome biogenesis, nucleolar stress response, nucleolar surveillance pathway, cancer, ribosomopathies, therapeutics

## Abstract

The nucleolus has emerged as a dynamic and multifunctional subnuclear organelle that integrates ribosome biogenesis with cellular growth and stress signalling. Dysregulation of nucleolar function is increasingly recognised as a central driver of human disease, linking altered ribosome production and nucleolar surveillance pathways to cancer, ribosomopathies, premature ageing syndromes, neurodegeneration, and immune disorders. In this review, we integrate structural, molecular, and disease-level perspectives on nucleolar biology to define how ribosome biogenesis and nucleolar surveillance regulate cellular state across physiological and pathological contexts, positioning the nucleolus as an active regulator of cell function. We outline areas of convergence, identify key unresolved questions, and highlight therapeutic vulnerabilities that arise, including opportunities for small-molecule inhibitors and gene-based approaches.

## 1. Introduction

The human nucleolus is a dynamic subnuclear compartment that integrates ribosome biogenesis (RiBi) with pathways governing cellular growth, stress responses, and homeostasis. While traditionally viewed solely as a site of ribosome production, the nucleolus is now recognised as a multifunctional organelle whose structure and activity are tightly coupled to cellular state. In addition to its role in RiBi, the nucleolus integrates transcriptional networks and the Nucleolar Surveillance Pathway, a stress-sensing mechanism activated by perturbations to nucleolar integrity that elicits responses ranging from cell cycle arrest and apoptosis to cellular preservation, thereby shaping genome function and cell fate.

Disruption of nucleolar function has profound biological consequences. Aberrant nucleolar function that drives dysregulated ribosomal DNA transcription supports proliferative demands in cancer, whereas impaired nucleolar output resulting in reduced ribosome production and defective nucleolar surveillance underlies a clinically heterogeneous group of disorders termed ribosomopathies. In these settings, nucleolar dysfunction is not simply a downstream consequence of disease but an active driver of pathology, positioning the nucleolus as a central yet incompletely understood regulator of cellular physiology and human disease. In this review, we synthesise current understanding of nucleolar function and its disruption in disease, highlighting therapeutic vulnerabilities that arise and emerging strategies to exploit them, including small-molecule inhibitors and gene-based approaches.

## 2. The Nucleolus

**Figure 1:**
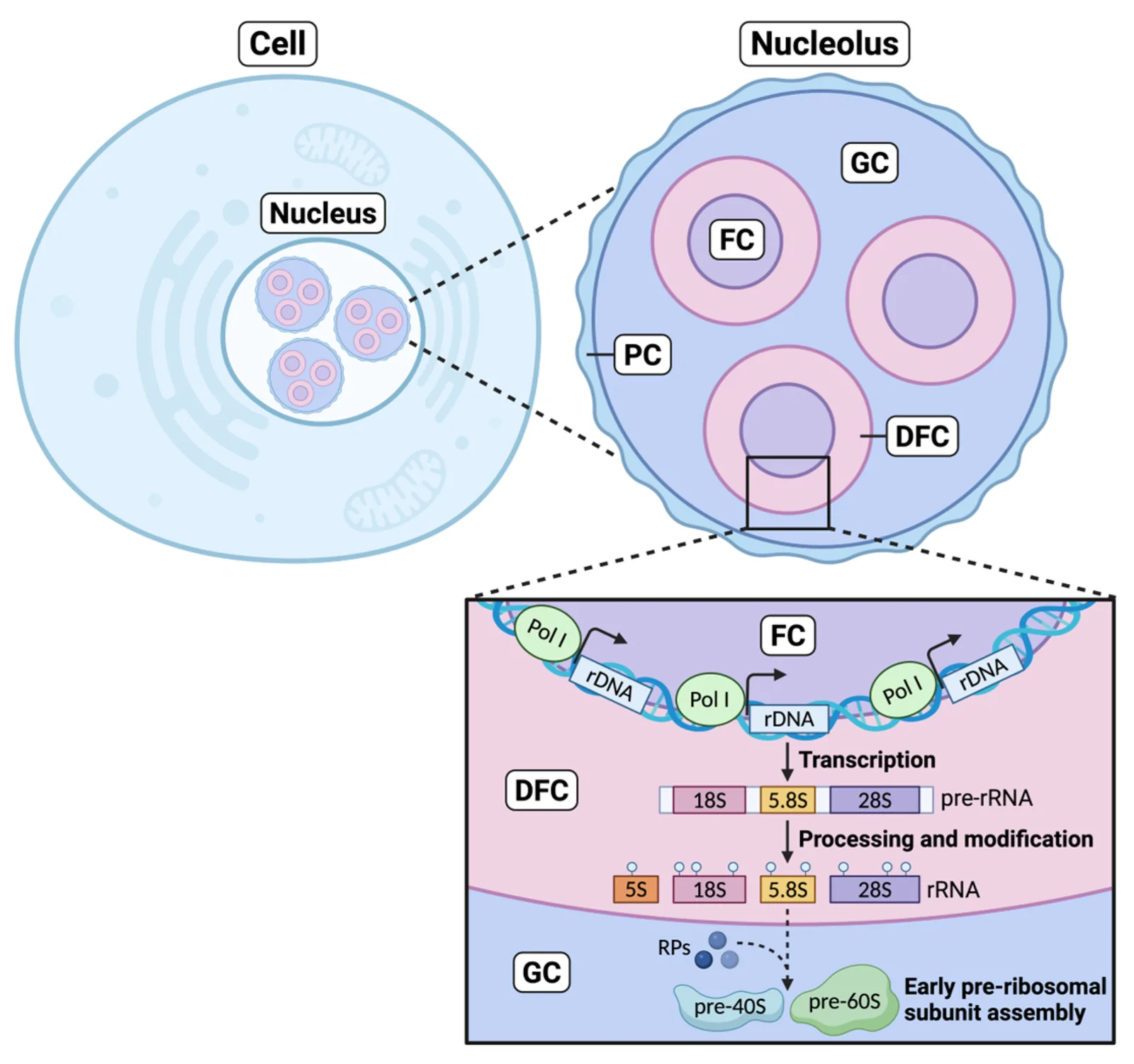
**Structure of the Nucleolus.** The nucleolus is a membrane-less organelle present within the nucleus of a cell. The nucleolus comprises three concentric sub-compartments: the innermost fibrillar centre (FC), the surrounding dense fibrillar component (DFC), and the outer granular component (GC), within which multiple FC-DFC modules are embedded. The GC is surrounded by perinucleolar chromatin (PC). RNA Polymerase I (Pol I)-mediated ribosomal DNA (rDNA) transcription occurs at the FC-DFC interface, after which nascent precursor ribosomal RNA (pre-rRNA) transcripts are processed and modified in the DFC and assemble with ribosomal proteins (RP) predominantly in the GC to form early pre-ribosomal subunits. Solid arrows indicate processes occurring primarily within the nucleolus, whereas dashed arrows indicate processes that continue beyond the nucleolus.

The human nucleolus is a dynamic, membrane-less subnuclear organelle that plays an essential role in RiBi and functions as a central hub coordinating cellular growth and stress signalling pathways. Nucleoli assemble around actively transcribed 47S ribosomal RNA (rRNA) genes, known as ribosomal DNA (rDNA), which are clustered in nucleolar organiser regions (NORs) on the short arms of the five acrocentric chromosomes in humans (13, 14, 15, 21, and 22) [1,2]. The nucleolus exhibits a highly organised structure comprising three immiscible concentric sub-compartments: the innermost fibrillar centre (FC), the surrounding dense fibrillar component (DFC), and the outer granular component (GC), within which multiple FC-DFC modules are embedded (Figure 1) [3,4]. The GC is encircled by perinucleolar chromatin (PC), a ring of condensed, transcriptionally inactive chromatin enriched in developmental and sensory perception genes that may also extend into the nucleolar interior [3,5,6]. This multilayered organisation arises through reversible liquid–liquid phase separation (LLPS), driven in part by the nucleolar proteins fibrillarin (FBL) and nucleophosmin (NPM1) [3,4,7]. Collectively, this organisation reflects a functional architecture in which spatial compartmentalisation is tightly coupled to sequential steps in rDNA transcription, rRNA processing, and ribosomal subunit assembly (Figure 1).

While the nucleolus is implicated in a broad range of cellular processes, this review outlines its canonical role in RiBi and stress signalling, both of which are implicated in the pathophysiology of diverse diseases.

## 3. The Nucleolus Coordinates Ribosome Biogenesis

### 3.1. Ribosome Biogenesis

#### 3.1.1. Transcription of rDNA

**Figure 2:**
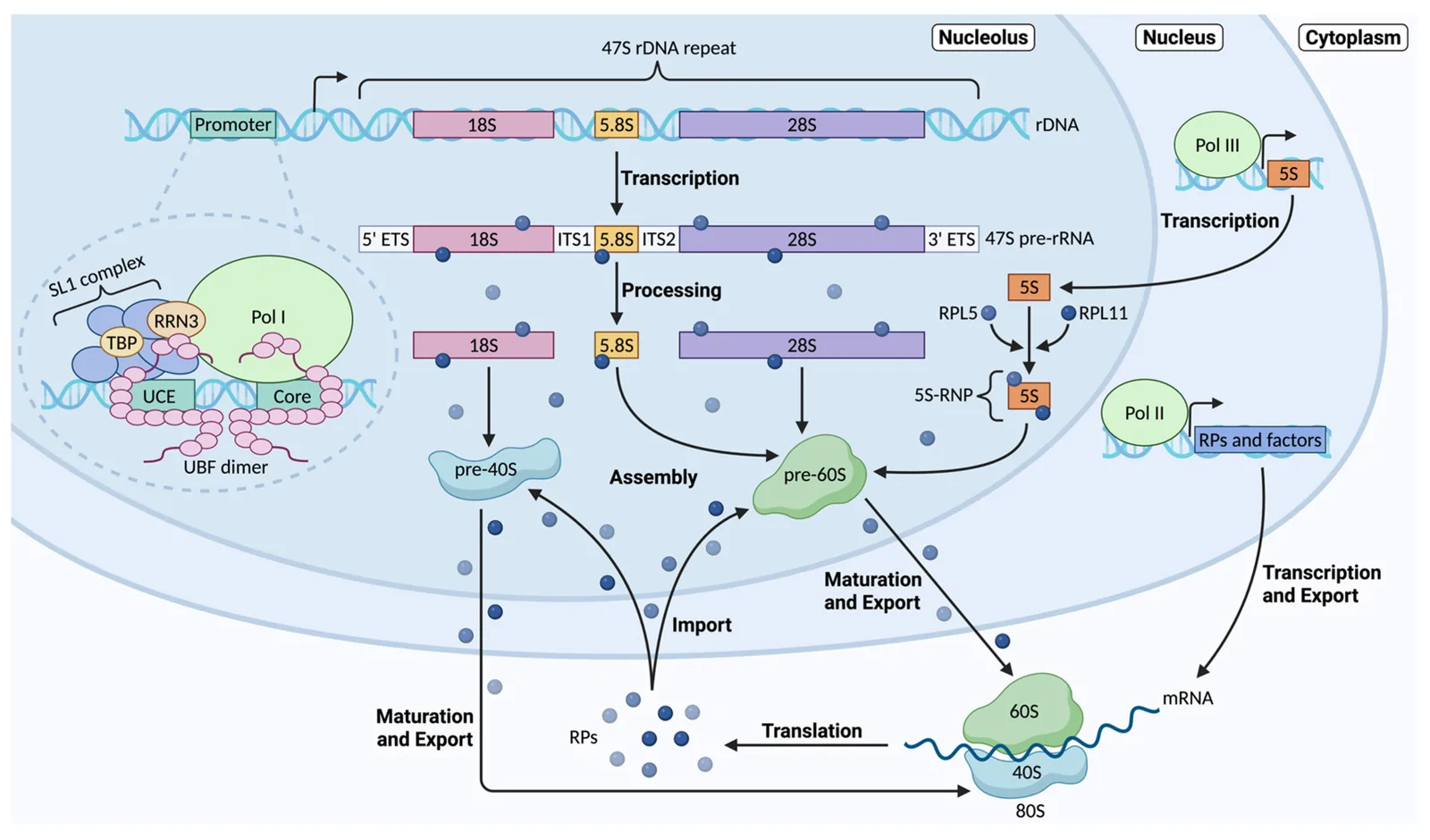
**The Process of Ribosome Biogenesis.** Ribosome biogenesis (RiBi) commences in the nucleolus around clusters of actively transcribed 47S ribosomal DNA (rDNA) repeats. The preinitiation complex (PIC), comprising the SL1 complex (consisting of TATA-binding protein (TBP) and TBP-associated factors), transcription initiation factor-IA (TIF-IA/RRN3), upstream binding factor (UBF), and additional transcription factors, assembles on the rDNA promoter, which contains an upstream control element (UCE) and core promoter. RNA Polymerase I (Pol I) transcribes the 47S precursor ribosomal RNA (pre-rRNA), which is subsequently processed to produce the 18S, 5.8S, and 28S rRNA. The 5S rRNA is transcribed by RNA Polymerase III (Pol III) in the nucleoplasm. Genes encoding ribosomal proteins (RPs) and RiBi factors are transcribed by RNA Polymerase II (Pol II) in the nucleoplasm and then translated into proteins in the cytoplasm. The RPs are then imported into the nucleus and nucleolus for assembly into their respective ribosomal subunits. The 5S rRNA complexes with RPL5 and RPL11 to form the 5S-ribonucleoprotein particle (5S-RNP). The small subunit RPs and 18S rRNA assemble to form the pre-40S subunit, and the large subunit RPs, 5.8S and 28S rRNA, and the 5S-RNP assemble to form the pre-60S subunit. These subunits are exported into the cytoplasm and undergo final maturation steps to produce the 40S and 60S subunits that subsequently form the mature 80S ribosome.

RiBi in mammalian cells (Figure 2) is spatially organised within the nucleolus, whose three sub-compartments coordinate successive steps of the process (Figure 1) [3,4]. It begins with transcription of 47S precursor rRNA (pre-rRNA) from rDNA repeats by RNA Polymerase I (Pol I) at the FC-DFC interface, where active Pol I complexes are enriched [3,4]. In contrast, the FC contains unengaged Pol I factors, while the GC contains inactive rDNA repeats [3,4]. These rDNA repeats, present at ~200–600 copies in the human genome, account for ~60% of total cellular transcription [8,9,10,11]. However, only a portion are actively transcribed at any time, with activity governed by upstream signalling and epigenetic state [12,13].

Structurally, human rDNA repeats are predominantly arranged in head-to-tail tandem arrays, with about one third inverted or palindromic [14]. Each repeat comprises a transcribed region followed by an intergenic spacer (IGS) with 5′ and 3′ terminator elements [10,12]. The transcribed region encodes the 47S pre-rRNA, which gives rise to 18S, 5.8S, and 28S rRNAs separated by internal transcribed spacers (ITS1, ITS2) and flanked by external transcribed spacers (5′ ETS, 3′ ETS) [10,12]. The IGS contains regulatory elements including the rDNA promoter, comprising an upstream control element (UCE) and core region, and spacer promoters upstream of enhancers [10,12].

Pol I transcription is initiated by binding of upstream binding factor (UBF) and the selectivity factor 1 (SL1) complex, composed of TATA-binding protein (TBP) and five Pol I-specific TBP-associated factors (TAFs), to the rDNA promoter, where the preinitiation complex (PIC) assembles (Figure 2) [15]. The PIC recruits Pol I via transcription initiation factor-IA (TIF-IA/RRN3), enabling stable Pol I-SL1 engagement and promoter escape [15].

#### 3.1.2. Pol II and Pol III Transcription and Pre-rRNA Processing

5S rRNA is transcribed by RNA Polymerase III (Pol III) from tandem repeats on the distal end of chromosome 1 in the nucleoplasm [16]. RNA Polymerase II (Pol II) transcribes genes encoding the ~80 ribosomal proteins (RPs), over 200 RiBi assembly factors, and over 200 small nucleolar RNPs (snoRNPs) in the nucleoplasm [17,18,19]. Pol II has been proposed to facilitate rRNA expression by regulating Pol I-dependent intergenic noncoding RNAs in mouse nucleoli [20]; however, whether this mechanism is conserved in humans remains unclear, with corresponding transcripts not detected in our analysis of human RNAseq data (unpublished observations). RPs and assembly factors are translated in the cytoplasm, then imported into the nucleus via importins and karyopherins recognising nuclear localisation signal (NLS) motifs, with nucleolar entry facilitated by nucleolar localisation signals (NoLS) [21,22,23].

Following 47S pre-rRNA transcription, nascent transcripts are sorted from the FC-DFC interface to the DFC, which is enriched in pre-rRNA processing and modification factors [3,4]. Pre-rRNAs associate co-transcriptionally with RPs and snoRNPs, undergoing folding, chemical modification (primarily 2′-O-ribose methylation and pseudouridylation catalysed by C/D and H/ACA box snoRNPs), and sequential endo- and exonuclease cleavage at multiple spacer sites to generate processing intermediates and, ultimately, the mature 5.8S, 18S, and 28S rRNAs [17,24,25].

#### 3.1.3. Ribosomal Subunit Assembly

Following rRNA processing, RiBi continues in the GC, which contains maturing ribosomal subunits and supports later stages of assembly [3,26]. Within the GC, RPs assemble stepwise with rRNAs to form pre-60S and pre-40S ribosomal subunits, with the latter maturing from a small subunit (SSU) processome [27,28,29]. In the nucleoplasm, 5S rRNA associates with RPL5 and RPL11 to form the trimeric 5S-ribonucleoprotein particle (5S-RNP), which assembles late onto maturing pre-60S subunits with the assistance of assembly factors, including regulator of ribosome synthesis 1 homolog (RRS1), brix domain-containing protein 1 homolog (BXDC1), and HEAT-repeat containing 3 (HEATR3), forming the central protuberance essential for ribosome function [30,31,32,33].

The pre-40S and pre-60S subunits are exported through the nuclear pore complex (NPC) and undergo final maturation in the cytoplasm to generate the mature 40S (small) and 60S (large) subunits, with some RPs assembling during both nuclear and cytoplasmic stages [29,34,35]. The mature 40S subunit contains ~33 RPs and 18S rRNA, and the 60S subunit comprises ~47 RPs along with 5S, 5.8S, and 28S rRNAs [36]. For translation initiation, the small subunit binds the 5′ end of mRNA and scans the 5′ untranslated region (UTR) until reaching the AUG start codon, where the 60S subunit joins to form the functional 80S ribosome [37]. During translation, the 40S subunit decodes codons via transfer RNA (tRNA) anticodons and the 60S subunit catalyses peptide bond formation via its peptidyl transferase centre, elongating the polypeptide chain [38]. As RiBi and translation are essential for cell growth and proliferation, yet highly energetically demanding processes, they are tightly regulated at multiple levels to match cellular metabolic state and proliferative demands, as discussed in the following sections.

### 3.2. Regulation of Ribosome Biogenesis

**Figure 3:**
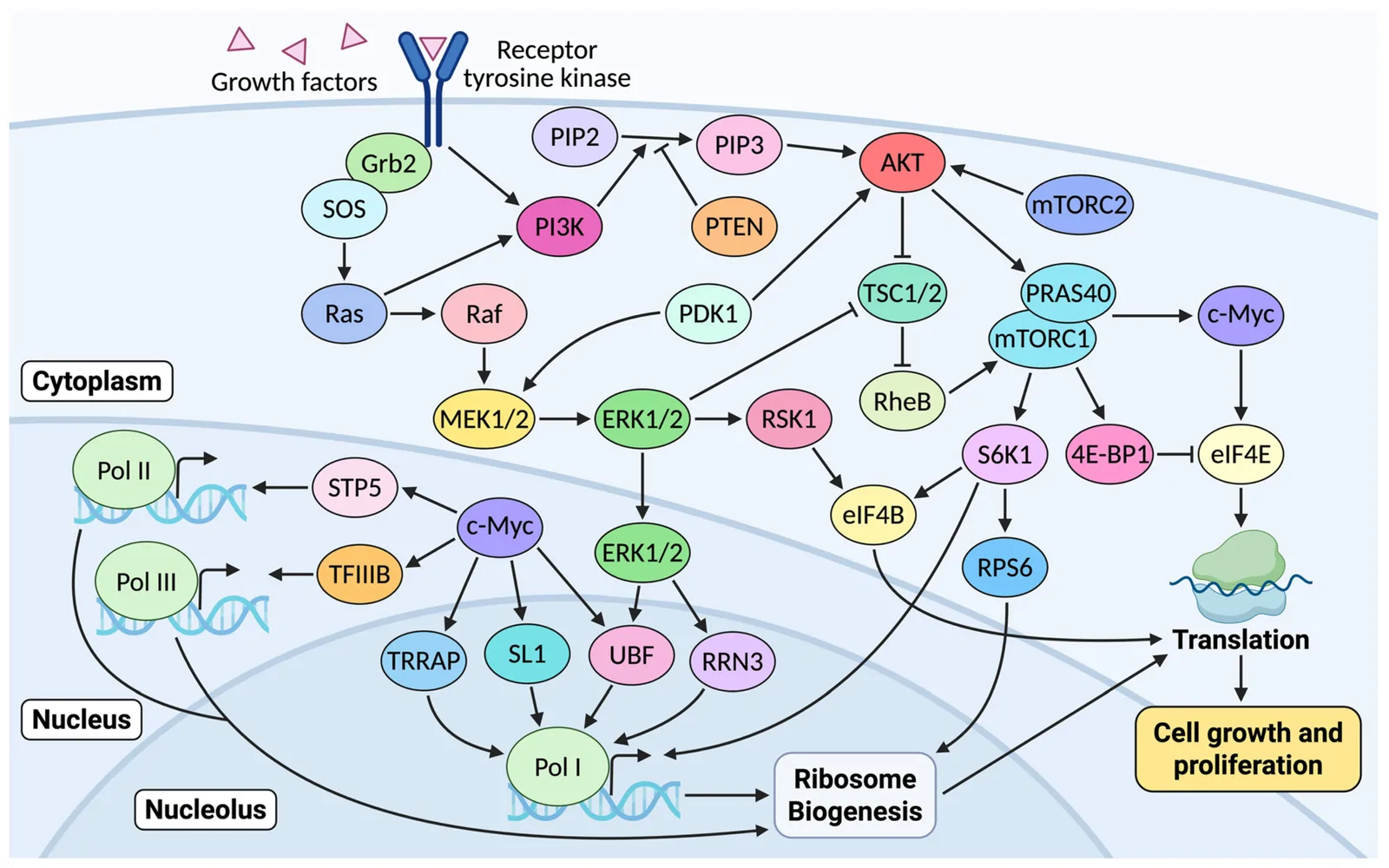
**Regulation of Ribosome Biogenesis by PI3K/AKT/mTORC1, MAPK, and MYC Signalling.** Growth factor receptor activation engages the PI3K/AKT/mTORC1 and MAPK pathways, and MYC (e.g., c-Myc) coordinates transcriptional output across all three RNA polymerases. Collectively, these pathways converge to regulate ribosomal DNA transcription, ribosomal protein production, and translation in response to nutrient availability and energy status, thereby coupling ribosome biogenesis to cellular growth, proliferation, and metabolic demands.

RiBi is the most energy-intensive cellular process and is therefore tightly regulated by cellular stress and nutrient availability, including amino acids, glucose, insulin, fatty acids, and growth factors, to balance energy expenditure with growth and proliferation [39,40]. These regulatory inputs are mediated through interconnected signalling pathways that couple environmental and cellular cues to nucleolar function. Multiple signalling networks, including PI3K/AKT/mTORC1, mitogen-activated protein kinase (MAPK), MYC, and CDK-cyclin complexes, integrate extracellular and intracellular signals to control RiBi at both transcriptional and translational levels, including through epigenetic regulation of rDNA activity [40,41]. These signalling pathways are summarised in Figure 3 and briefly discussed below.

#### 3.2.1. The PI3K/AKT/mTORC1 Pathway

The PI3K/AKT/mTORC1 pathway is activated by growth factor receptor tyrosine kinases (RTKs), which stimulate class 1A phosphoinositide 3-kinases (PI3Ks) to convert phosphatidylinositol 4,5-bisphosphate (PIP_2_) to phosphatidylinositol 3,4,5-trisphosphate (PIP_3_), a process antagonised by phosphatase and tensin homolog (PTEN) [42,43]. PIP_3_ recruits AKT to the plasma membrane, where it is activated by phosphorylation at Thr308 by phosphoinositide-dependent protein kinase 1 (PDK1) and at Ser473 by mammalian target of rapamycin (mTOR) complex 2 (mTORC2) [44,45]. Activated AKT phosphorylates tuberous sclerosis protein 2 (TSC2), leading to activation of RheB, and phosphorylates proline-rich Akt substrate of 40kDa (PRAS40), relieving mTORC1 inhibition, thereby activating mTORC1 [46,47]. Activated mTORC1 subsequently phosphorylates ribosomal protein S6 kinase 1 (S6K1), which phosphorylates ribosomal protein S6 (RPS6), and eukaryotic translation initiation factor 4E binding protein 1 (4E-BP1) to promote RiBi, protein synthesis, and cell growth [48,49,50]. Through these effectors, the PI3K/AKT/mTORC1 pathway links growth factor signalling to ribosome production and translational output, ensuring that cellular protein synthesis capacity is matched to nutrient availability and growth demands.

#### 3.2.2. The MAPK Pathway

MAPKs convert extracellular signals into intracellular responses, with extracellular signal-regulated kinase 1/2 (ERK1/2) being among the best characterised [51]. ERK1/2 is activated by RTKs, which recruit growth factor receptor-bound protein 2 (Grb2) and Son of Sevenless (SOS) to activate Ras [52]. Ras then activates Raf, which phosphorylates MEK1/2, leading to ERK1/2 activation [51]. Activated ERK1/2 translocates from the cytoplasm to the nucleus, where it phosphorylates UBF and RRN3 to promote rRNA synthesis, and phosphorylates ribosomal S6 kinase 1 (RSK1), which activates eukaryotic translation initiation factor 4B (eIF4B) to promote translation initiation [53,54,55]. This pathway therefore directly links mitogenic signalling to activation of Pol I transcription and RiBi. While the canonical ERK1/2, p38, and JNK pathways are well characterised, emerging evidence suggests that atypical MAPKs, including ERK3, ERK4, ERK7, ERK8, and Nemo-like kinase (NLK), also contribute to the regulation of cell growth and proliferation, with potential implications in cancer progression and immune signalling, highlighting additional layers of complexity within MAPK signalling networks [56,57,58].

#### 3.2.3. MYC Regulation

The *MYC* proto-oncogene family, comprising *MYC* (c-Myc), *MYCN* (N-Myc), and *MYCL* (L-Myc), encodes transcription factors that control key cellular processes, including growth, differentiation, proliferation, cell cycle, DNA replication and repair, metabolism, and RiBi [59,60,61,62]. c-Myc is commonly hyperactivated in cancer, driving uncontrolled growth in part through increased ribosome production [63,64]. c-Myc promotes RiBi by coordinating transcription by all three RNA polymerases [65]. c-Myc enhances Pol I-mediated rRNA transcription by dimerising with *MYC*-associated factor X (MAX) to bind rDNA promoters, recruiting transformation/transcription domain-associated protein (TRRAP) and stimulating histone H3 and H4 acetylation to promote an open chromatin state [66,67,68]. c-Myc also complexes with transcription factor IIIB (TFIIIB) independently of MAX, recruiting lysine acetyltransferase 2A (KAT2A) and TRRAP to promote histone H3 acetylation, enhancing Pol III-mediated 5S rRNA and tRNA transcription [68,69]. For Pol II, c-Myc interacts with cyclin-dependent kinase 9 (CDK9) and recruits transcription elongation factor SPT5, promoting transcription of RP and RiBi factor genes [70,71]. In particular, c-Myc drives transcriptional upregulation of multiple core components of the Pol I transcriptional machinery, a level of coordinated regulation that is not observed for Pol II- or Pol III-associated components [65]. Through this integrated control of Pol I, II, and III, c-Myc functions as a central and uniquely positioned regulator of RiBi, coordinating both rDNA transcription and the biosynthetic capacity required to sustain ribosome production, particularly in proliferative and oncogenic contexts.

#### 3.2.4. CDK-Cyclin-Dependent Regulation

CDK-cyclin complexes coordinate RiBi with cell cycle progression by phosphorylating key transcription and processing factors [41]. During G1, CDK2-cyclin E phosphorylates RRN3 at Ser44, stimulating Pol I transcription [72]. UBF is phosphorylated at Ser484 by CDK4-cyclin D1 and CDK2-cyclin E during G1, and at Ser388 by CDK2-cyclin A and E during S/G2, strengthening its interaction with Pol I and promoting rRNA synthesis [73,74,75]. ERK signalling further enhances Pol I activity during G1 and S phase through activation of CDK4 and CDK6 [76]. Furthermore, CDK9-mediated phosphorylation of Treacle promotes Pol I recruitment to rDNA [77]. In contrast, RiBi is suppressed during mitosis through CDK1-dependent phosphorylation of nucleolar proteins [41,78,79]. During mitosis, phosphorylation of UBF and CDK1-cyclin B-mediated phosphorylation of the SL1 subunit TAF1-110 at Thr852 inhibit rDNA transcription [80,81,82]. Several pre-rRNA processing factors, including NPM1, MPP10, and MPP6, are also phosphorylated during mitosis, although the functional consequences of these modifications remain unclear [41,83,84,85]. Upon mitotic exit, dephosphorylation by PP1 and PP2A restores nucleolar protein activity and reinitiates RiBi [79].

#### 3.2.5. Epigenetic Regulation of rDNA Transcription

Although rDNA repeats are the most highly transcribed regions of the mammalian genome, a portion remain transcriptionally silenced, governed predominantly by epigenetic mechanisms that respond to energy status [12,13,86]. Active rDNA adopts a euchromatic, hypomethylated state enriched in active histone modifications upstream of enhancer regions, including the H2A.Z variant, H3 and H4 acetylation, and H3K4 methylation, whereas silent rDNA exhibits CpG hypermethylation alongside repressive modifications in the promoter, such as H4K16 and H4K20 deacetylation, H3K9 methylation, and H3K27me3 [12,13,87,88]. The switch between active and silent is driven primarily by three rDNA-silencing complexes. The nucleolar remodelling complex (NoRC), comprising SNF2h and TIP5, recruits the noncoding promoter-associated RNA (pRNA), DNMTs, and histone-modifying enzymes to block assembly of the PIC at the rDNA promoter [89]. The energy-dependent nucleolar silencing complex (eNoSC), composed of SUV39H1, NML, and SIRT1, silences rDNA in response to glucose starvation, protecting cells from energy-deprivation-induced apoptosis [90,91]. The nucleosome remodelling and deacetylation complex (NuRD), containing HDAC1/2, CHD3/4, GATAD2A/2B, RBBP7/4, MTA1/2/3, and MBD2/3, is activated by reduced cell growth and establishes a “poised” chromatin state that maintains silencing [92]. These complexes act within a broader, interdependent network of histone- and DNA-modifying proteins that reinforce rDNA transcriptional repression [12,13,86]. Beyond transcriptional regulation, rDNA silencing preserves genome stability by limiting aberrant recombination between sister chromatids [13,93]. Accordingly, its disruption is implicated in genome instability and, consequently, ageing and disease, including cancer and developmental, haematopoietic, autoimmune, and neurological disorders [94].

## 4. The Nucleolar Proteome

### 4.1. Early Mass Spectrometry Mapping of the Nucleolar Proteome

#### 4.1.1. Redefining the Nucleolus: Multifunctionality Beyond Ribosome Biogenesis

For much of the twentieth century, the nucleolus was viewed primarily as the site of RiBi. This view was challenged in 1998 when Thoru Pederson proposed the concept of a “plurifunctional nucleolus”, suggesting that the nucleolus supports processes beyond ribosome production [95]. This shift reframed the nucleolus as a dynamic regulatory hub rather than a structure dedicated solely to ribosome synthesis. The first efforts to define the nucleolar proteome began in 2002 with two independent mass spectrometry (MS)-based proteomic analyses of purified human nucleoli from HeLa cells, published by Andersen et al. and Scherl et al. [96,97]. These studies identified ~350 nucleolar-associated proteins and provided the first systematic molecular definition of nucleolar composition [96,97]. As expected, RPs and additional RiBi factors constituted a substantial fraction of the datasets, consistent with the canonical role of the nucleolus [96,97]. However, a large proportion of identified proteins were previously uncharacterised or not known to localise to the nucleolus, including factors involved in translation and mRNA metabolism [96,97]. Scherl et al. cautioned that the translation-associated proteins may have co-purified artefactually, or, alternatively, that these proteins may localise to nucleoli transiently or at low abundance [97]. Nonetheless, the proteomic analyses provided early molecular support for the plurifunctional model and indicated that nucleolar activity extends beyond RiBi. The studies also provided evidence that the nucleolus responds to transcriptional and metabolic perturbation [96], with further studies supporting the concept that nucleolar organisation is not solely dependent on active rDNA transcription [98].

#### 4.1.2. Limitations of Early Proteomic Approaches

Despite their impact, these fractionation-based proteomic studies have important limitations. Several well-established nucleolar proteins, including SL1 subunits, Gemin4, SRP68, and TERT, were not detected [96,97]. Additionally, of the ~700 proteins reported across these studies [96,97,98], only ~266 had experimental Gene Ontology validation for nucleolar localisation at the time of publication [99]. These discrepancies likely reflect technical constraints of the approach. Proteins that associate transiently or weakly with the nucleolus may dissociate during purification, while low-abundance or structurally atypical proteins may fall below detection thresholds or evade identification [100]. Conversely, contaminants may co-purify, reflecting the membrane-less nature of the nucleolus [100]. Further limitations arise from the use of a single cancer cell line, asynchronous cells, and a narrow range of cellular perturbations, which are unlikely to capture the full extent of context-dependent proteomic variation and cell cycle-specific dynamics. This is particularly important given evidence that nucleolar function is dysregulated in cancer (Section 6.1) and closely linked to cell cycle control [101]. To address these limitations, subsequent MS-based studies expanded the scope to additional cell types and physiological contexts, identifying 872 nucleolar proteins in Jurkat T cells [102], 504 nucleolar proteins in developing rat cerebral cortex [103], many of which were associated with human neurodevelopmental disorders, and >4500 proteins in human nucleoli from diverse cell types under various conditions [104]. Together, these studies establish the nucleolus as a highly dynamic and context-dependent proteomic environment, reinforcing its role as a central integrator of cellular state.

### 4.2. The Human Protein Atlas as a Resource for the Nucleolar Proteome

More recently, insights into the nucleolar proteome have emerged from the Human Protein Atlas (HPA), which provides imaging-based protein subcellular localisation across 64 cell lines expressing ~98% of human protein-coding genes, providing broad proteome coverage [105]. Using HPA data, Stenström et al. in 2020 performed the first spatiotemporal mapping of the human nucleolar proteome across sub-compartments and cell cycle stages, identifying 1318 nucleolar proteins, of which 1031 localised to the whole nucleolus, 287 to the FC/DFC, and 157 to a distinct nucleolar rim [5]. Based on its distinct proteomic composition, this rim was proposed to represent a fourth nucleolar sub-compartment [5]. Over 87% of nucleolar proteins also localised to other cellular compartments, most commonly the nucleoplasm, cytosol, and mitochondria, indicating functions beyond RiBi [5]. Compared with datasets from Anderson et al. and Scherl et al., 550 proteins were not observed in the HPA dataset, while 1009 proteins were uniquely identified, including 292 not expressed in HeLa cells, highlighting the complementarity and cell-type dependence of these datasets [5].

Stenström et al. further characterised nucleolar proteome dynamics throughout the cell cycle with a single-cell imaging approach, an aspect not resolved by bulk MS approaches [5]. They identified 65 nucleolar proteins that re-localised during cell division to the chromosomal periphery, an RNA-protein sheath covering the outer surface of mitotic chromosomes, including 36 not previously reported at mitotic chromosomes, and 19 recruited late in mitosis, suggesting potential roles in nucleolar reassembly [5]. Proteins that relocated during mitosis were enriched in intrinsically disordered regions, consistent with roles in phase separation and condensate dynamics [5].

### 4.3. Emerging Strategies for Defining the Nucleolar Proteome

Immunofluorescence (IF)-based approaches offer several key advantages over MS-based approaches, including detection of low-abundance proteins, single-cell resolution, visualisation across the cell cycle, and characterisation of multi-compartment localisation, which is particularly important given the dynamic exchange of nucleolar components with the nucleoplasm [3]. However, the HPA dataset is limited by potential false positives from non-specific antibodies, incomplete localisation validation by knockdown or independent antibodies, fixation or staining artefacts, and computational classification of sub-compartments rather than marker-based experimental validation. Accordingly, imaging- and MS-based approaches are complementary for defining nucleolar proteome organisation and dynamics.

Beyond defining spatiotemporal information with imaging-based approaches, functional understanding of the nucleolus also requires mapping protein–protein interactions, as many nucleolar processes, including biochemical activity, biomolecular sequestration, and condensate formation, are mediated by dynamic interaction networks [3]. However, conventional nucleoli isolation is poorly suited to capturing transient or weakly associated nucleolar components, while also being labour-intensive and prone to sample degradation [100]. To overcome these limitations, proximity-labelling technologies have recently emerged to enable identification of nucleolar protein interaction networks, providing new insights into nucleolar biology.

Emerging technologies include DenseMAP, which resolved a GC-specific proteome and revealed insights into nucleolar protein quality control and stress responses [106]; light-induced targeting of endogenous condensates (LiTEC) combined with proximity-based biotinylation (BioID) to profile condensate protein interactomes [107]; ChIP-MS targeting RPA194 to identify rDNA-associated proteins in breast cancer [108]; and nucleolar beacon (NoB), which enabled analysis of nucleolar interactions in living cells without fractionation-based purification [109]. Collectively, these approaches provide powerful platforms for defining nucleolar interaction networks with temporal and contextual resolution.

Despite substantial progress, a single, definitive nucleolar proteome has yet to be defined. Instead, the nucleolus comprises overlapping, context-dependent proteomes influenced by cell type, developmental stage, physiological state, and stress conditions. Accordingly, integrating proximity-labelling, imaging, and MS-based approaches will be required to define how nucleolar composition is specified and remodelled across biological contexts.

### 4.4. Expanding Nucleolar Functions Beyond Ribosome Biogenesis

While many nucleolar proteins identified in proteomic studies remain functionally uncharacterised, leaving nucleolar functions incompletely understood, these datasets have nonetheless redefined the nucleolus from a structure dedicated to RiBi to a multifunctional regulatory hub. It is now implicated in diverse cellular processes, including responses to viral and bacterial infection [110,111], cell cycle regulation [112,113], assembly of non-ribosomal ribonucleoprotein (RNP) complexes [114], small interfering RNA (siRNA) regulation [115], non-ribosomal RNA transcription [116], telomere maintenance [117,118], the nucleolar DNA damage response (n-DDR) for repair of damaged rDNA [119,120,121,122], and coordination of cellular stress signalling [11,32,123].

Within this broader functional landscape, this review places a focus on nucleolar stress signalling, reflecting its broad implications for cell fate and disease, while recognising that it represents one of several key functional outputs of the nucleolus.

## 5. The Nucleolar Surveillance Pathway

Concurrent with the first nucleolar proteomic studies, Rubbi and Milner established that the nucleolus functions as a cellular stress sensor, monitoring disruptions in RiBi and directing cell fate decisions [123]. They provided the first evidence that inhibition of Pol I transcription activates p53 independently of DNA damage, leading to p53-dependent cell cycle arrest [123].

Further studies have shown that this stress sensor mechanism, termed the “Nucleolar Surveillance Pathway” (NSP; or Nucleolar Stress Response), is initiated by stressors that disrupt nucleolar integrity. These include perturbations to rRNA transcription or processing, ribosome assembly or export, and rDNA damage (collectively referred to as ribosomal stress), as well as nutrient or serum starvation, cell contact inhibition, pH changes, ultraviolet radiation, viral infection, aberrant protein accumulation, impaired transcription or translation, hypoxia, reactive oxygen species (ROS) accumulation, non-ribosomal DNA damage, and oxidative, osmotic, thermal, oncogenic, or metabolic stress [11,32,119,123,124,125,126].

Several of these stressors, including nutrient starvation, oncogenic signalling, and metabolic stress, act primarily through the RiBi-regulatory pathways discussed in Section 3.2. DNA damage and ROS accumulation, by contrast, act directly at the level of rDNA integrity, disrupting nucleolar organisation through distinct mechanisms. rDNA repeats are particularly susceptible to genomic insult due to their high copy number, repetitive structure, and high transcriptional activity [119,121,127]. DNA damage at rDNA loci can trigger the n-DDR [119,120,121,122], providing a route into the NSP distinct from DNA damage-independent activation. ROS accumulation similarly damages rDNA, activating the n-DDR and converging on this same route to p53 stabilisation [128].

In response to rDNA damage, the n-DDR transiently inhibits Pol I transcription and reorganises the nucleolus into nucleolar “caps” to facilitate repair [119,121,122,129]. Within the nucleolus, Treacle (TCOF1) is a central regulator of this repair process, recruiting the MRE11-RAD50-NBS1 (MRN) complex and TOPBP1 to damaged rDNA following APE1-mediated ataxia telangiectasia mutated (ATM)/ataxia telangiectasia and Rad3 related (ATR) activation [122,130,131,132,133,134,135]. This is exemplified in the ribosomopathy Treacher Collins Syndrome (TCS), in which TCOF1 dysfunction is linked to elevated DNA damage in model systems [136,137,138,139].

Given the vulnerability of rDNA loci to DNA damage, several global DDR factors also localise to the nucleolus and contribute to n-DDR signalling, with additional effectors continuing to be identified [120]. For example, poly(ADP-ribose) polymerase 1 (PARP1) associates with the NoRC subunit TIP5 to silence rDNA transcription and interacts with the RecQ helicases Bloom syndrome protein (BLM) and Werner syndrome helicase (WRN) [140,141,142]. These helicases support Pol I transcription while also resolving topological and replication-associated stress at rDNA loci, indicative of a dual functional role [143,144,145]. As n-DDR defects promote rDNA instability and cancer progression [120], further elucidating their mechanisms and crosstalk with global DNA repair may reveal how nucleolar surveillance coordinates DNA damage responses, while also identifying potential therapeutic targets.

Depending on the duration and severity of the insult, the NSP can trigger responses ranging from apoptosis to cellular preservation, thereby playing a critical role in maintaining genomic integrity [11,125]. NSP activation primarily stabilises the transcription factor and tumour suppressor protein p53 (*TP53*), termed the p53-dependent NSP, although p53-independent NSP responses have also been described, both of which are discussed in the following sections.

### 5.1. p53-Dependent Nucleolar Surveillance Pathways

#### 5.1.1. The Ribosomal Protein-MDM2-p53 Axis

**Figure 4:**
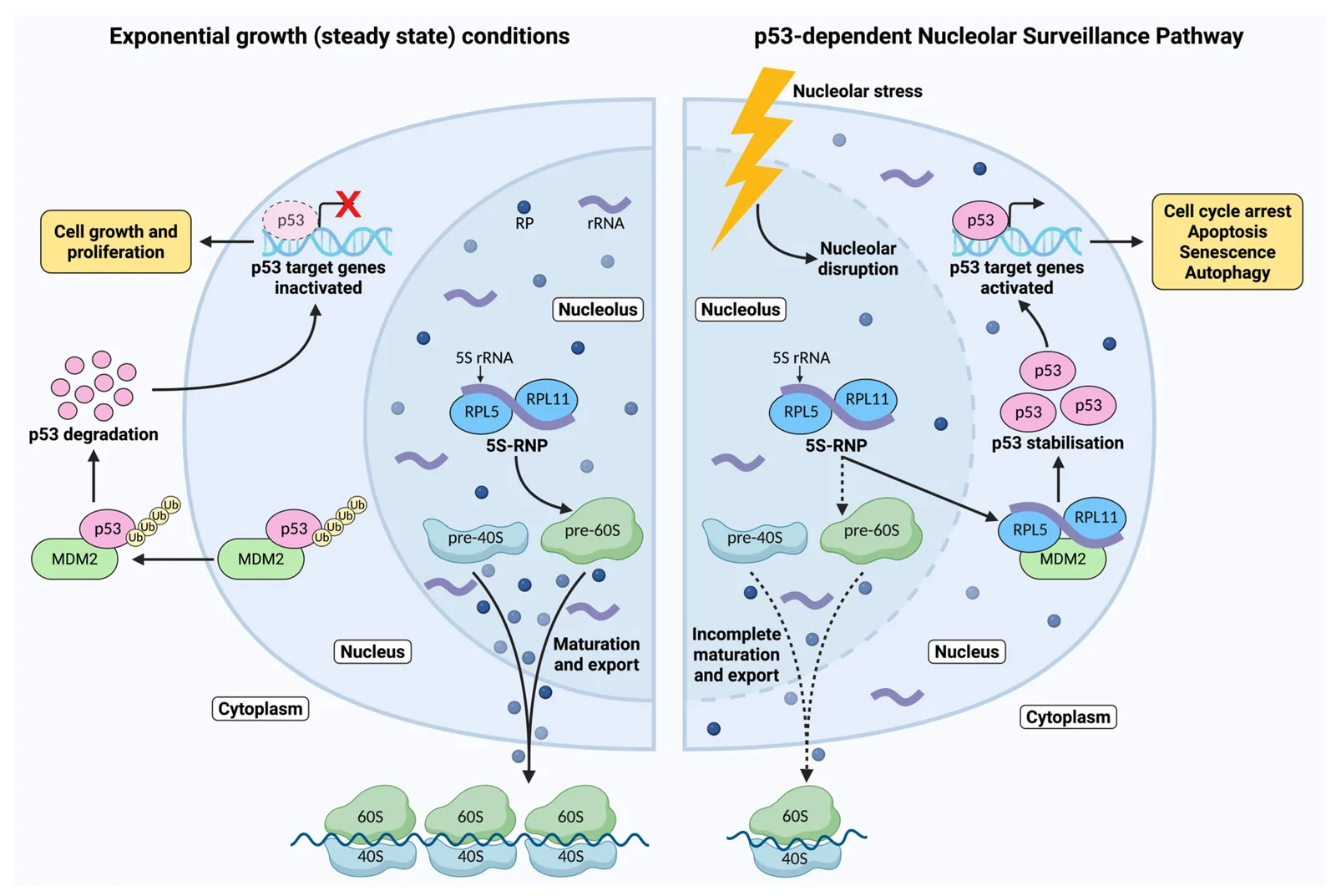
**The Canonical p53-Dependent Nucleolar Surveillance Pathway.** Under exponential growth (steady state) conditions, ribosomal proteins (RPs) and ribosomal RNA (rRNA) are integrated into newly synthesised ribosomes. The 5S-ribonucleoprotein particle (5S-RNP) complex, consisting of RPL5, RPL11, and 5S rRNA, is incorporated into the pre-60S subunit. MDM2 (an E3 ubiquitin ligase) ubiquitinylates p53, targeting it for proteasomal degradation, thereby maintaining low intracellular levels of p53 and suppressing transcription of p53 target genes. In response to nucleolar-acting stress and disruption, the 5S-RNP and other RPs and rRNA are no longer incorporated into the pre-40S and pre-60S subunits and exist in the nucleoplasm in “free” form. As incorporation of the 5S-RNP into the pre-60S subunit is impaired (dashed arrows), unincorporated 5S-RNP instead binds to MDM2, which prevents the ubiquitin-mediated proteasomal degradation of p53. This leads to the stabilisation of p53 protein. As a result, p53 induces transcription of its target genes, activating pathways that result in cell cycle arrest, apoptosis, senescence, and/or autophagy.

Activation of the NSP stabilises p53 primarily through a 5S-RNP-dependent mechanism (Figure 4). Under exponential growth conditions, p53 levels are regulated by the E3 ubiquitin ligase mouse double minute 2 (MDM2), which poly-ubiquitinates p53, targeting it for 26S proteasomal degradation [30,32,146,147,148,149]. NSP activity disrupts RiBi, causing ribosomal components, particularly the 5S-RNP, to accumulate in a “free” nucleoplasmic pool [30,32,146,147,148,149]. The 5S-RNP binds the central acidic domain of MDM2, inhibiting its activity and preventing p53 ubiquitination and degradation, resulting in p53 accumulation [30,32,146,147,148,149]. Stabilised p53 then induces transcription of its downstream effectors, promoting cell cycle arrest, apoptosis, senescence, or autophagy depending on the cellular context [30,32,146,147,148,149,150].

In addition to the 5S-RNP, multiple RPs contribute to NSP signalling by binding and inhibiting MDM2 in a context-dependent manner [151,152,153,154,155,156,157,158,159,160,161,162,163,164,165]. Additionally, 5S rRNA can inhibit the MDM2 homolog MDMX (MDM4), which suppresses p53 activity and enhances MDM2 function despite lacking intrinsic E3 ubiquitin ligase activity [166]. Among the RPs, RPL5 and RPL11, as components of the 5S-RNP, are essential for p53 activation, whereas additional RPs likely provide functional redundancy and context-specific modulation of the pathway [30,32,146,147,148,149].

The pathway is further regulated by feedback loops. *MDM2* is a p53 transcriptional target, ensuring that p53 stabilisation induces *MDM2* expression and subsequent p53 downregulation [147,150]. Additionally, p53 suppresses transcription of several RPs, including RPS27L and RPS25, restricting the pool of MDM2-interacting RPs [151,155,167]. Several RPs, including RPS7, RPL6, RPL26, and RPS27-like (RPS27L), are also direct substrates for MDM2-mediated degradation, further limiting their capacity to sustain p53 activation [151,165,168,169]. Thus, RPs both regulate and are regulated by the MDM2-p53 axis.

#### 5.1.2. Additional Pathways of p53 Stabilisation

**Figure 5:**
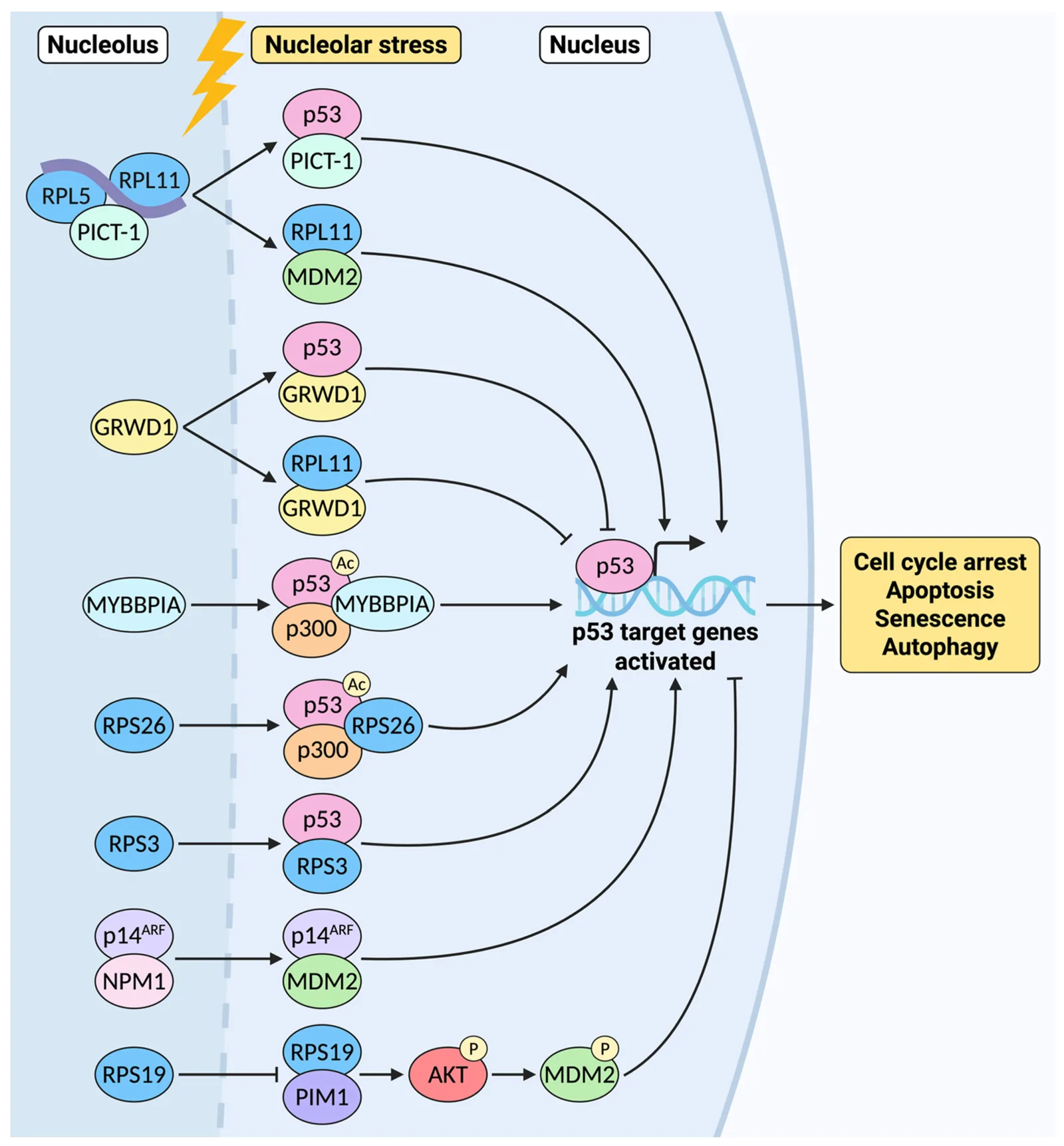
**Additional p53-Dependent Nucleolar Surveillance Pathways.** Beyond the canonical ribosomal protein-MDM2-p53 axis, nucleolar stress engages multiple parallel pathways to regulate activation of p53 transcriptional targets. These include pathways mediated by PICT-1, GRWD1, MYBBP1A, RPS26, RPS3, NPM1/p14^ARF^, and RPS19-PIM1-AKT-MDM2 signalling, each operating under distinct nucleolar stressors and cellular contexts.

While the RP-MDM2-p53 axis is the primary driver of p53 stabilisation, additional pathways also exist and contribute to this response (outlined below and summarised in Figure 5).

**PICT-1:** RPL11 promotes p53 stabilisation through tumour suppressor protein interacting with carboxyl terminus 1 (PICT-1), which stabilises PTEN and functions as a 60S subunit biogenesis factor [170,171]. Under exponential growth conditions, PICT-1 retains RPL5 and RPL11 within the nucleolus through direct interaction with the 5S-RNP [30]. Upon DNA damage-induced nucleolar stress, PICT-1 is degraded, enabling RPL11 translocation into the nucleoplasm to enhance p53 stabilisation and apoptosis [172,173,174]. PICT-1 can also directly stabilise p53 under ribosomal stress, highlighting its dual role in coordinating RiBi and stress signalling [175].

**GRWD1:** Glutamate-rich WD repeat containing-1 (GRWD1), a chromatin regulator overexpressed in multiple cancers, inhibits p53 activation through RPL11 [176,177,178]. Stress-induced GRWD1 translocation to the nucleoplasm disrupts the RPL11-MDM2 interaction, suppressing p53 activation, while GRWD1 can also bind the p53 DNA-binding domain to modulate transcriptional activity [179,180,181].

**MYBBP1A:** MYB-binding protein-1A (MYBBP1A), which is normally nucleolar through rRNA binding, relocalises to the nucleoplasm upon stress, where it cooperates with RPL5 and RPL11 to enhance p53 acetylation through p300 recruitment [182,183,184].

**RPS26, RPL26, and RPS3:** RPS26 enhances p53 acetylation by complexing with p53 and p300 following DNA damage, RPL26 binds the 5′ UTR of *TP53* mRNA to promote translation after DNA damage, and RPS3 protects p53 from MDM2-mediated ubiquitination [154,159,185]. Together, these observations indicate that RPs regulate p53 not only through MDM2 inhibition but also at the level of translation and post-translational modification.

**NPM1, p14^ARF^, and nucleolar regulators:** Under basal conditions, NPM1 stabilises alternative reading frame protein (p19^ARF^ in mouse, p14^ARF^ in human) in the nucleolus [186]. Upon stress, p14^ARF^ relocates to the nucleoplasm and binds MDM2 to inhibit its activity, while NPM1 also modulates p53-MDM2 interactions in the nucleus [186,187,188]. NPM1 and p14^ARF^ form feedback loops involving MDM2, and their dysregulation can suppress p53 signalling [189,190]. Additional nucleolar proteins, including SRSF1, PAK1IP1, CSIG, and nucleostemin, also interact with MDM2 under stress [191,192,193,194].

**AKT-MDM2 regulation:** Pim-1 proto-oncogene, serine/threonine kinase (PIM1) phosphorylates AKT at Ser473, enabling AKT-mediated phosphorylation and activation of MDM2 to promote p53 degradation [195,196]. RPS19 depletion destabilises PIM1, reducing AKT phosphorylation and inactivating MDM2, thereby stabilising p53 [196,197].

**NCL:** NCL modulates p53 expression by regulating *TP53* mRNA translation and relocalising under stress to influence replication-associated processes [185,198,199].

**ASK1/p38 signalling:** Ribosomal stress activates apoptosis signal-regulating Kinase 1 (ASK1)/p38 signalling to promote p53 phosphorylation and G1 arrest [200].

Collectively, these studies demonstrate that p53 stabilisation is governed by a multilayered network extending beyond the RP-MDM2 axis, integrating translational, post-translational, and signalling-based inputs. A key unresolved question is how these pathways are prioritised and coordinated across different stress contexts to determine p53-dependent cell fate outcomes.

#### 5.1.3. Mechanistic Determinants and Stress Specificity of the p53-Dependent Nucleolar Surveillance Pathway

Despite extensive study, the mechanisms underlying the p53-dependent NSP remain incompletely resolved, particularly the factors required for activation. To address this, a functional genomic screen conducted by our group identified components required for NSP-mediated p53 stabilisation in response to nucleolar stress induced by RPS19 depletion [32]. The screen confirmed the essential roles of RPL5 and RPL11, whilst also identifying several new candidates, most notably *HEATR3*, a factor essential for 60S subunit maturation and 5S-RNP biogenesis, whose biallelic loss-of-function variants are associated with Diamond–Blackfan Anaemia Syndrome (DBAS) [32,33]. In contrast, several previously reported regulators of p53 stabilisation, including PICT-1, MYBBP1A, NPM1, and SRSF1, were not identified as essential NSP components, suggesting tissue- or context-specific roles [32]. To assess the breadth of stressors that activate the p53-dependent NSP, cells were exposed to diverse genetic, physiological, and pharmacological insults [32]. Most nuclear and genetic perturbations stabilise p53 through the 5S-RNP-MDM2 axis, whereas proteotoxic stressors acted independently of this pathway [32]. These findings support Rubbi and Milner’s hypothesis that the nucleolus functions as a universal stress sensor that maintains p53 homeostasis in response to diverse cellular perturbations [32,123].

### 5.2. p53-Independent Nucleolar Surveillance Pathways

**Figure 6:**
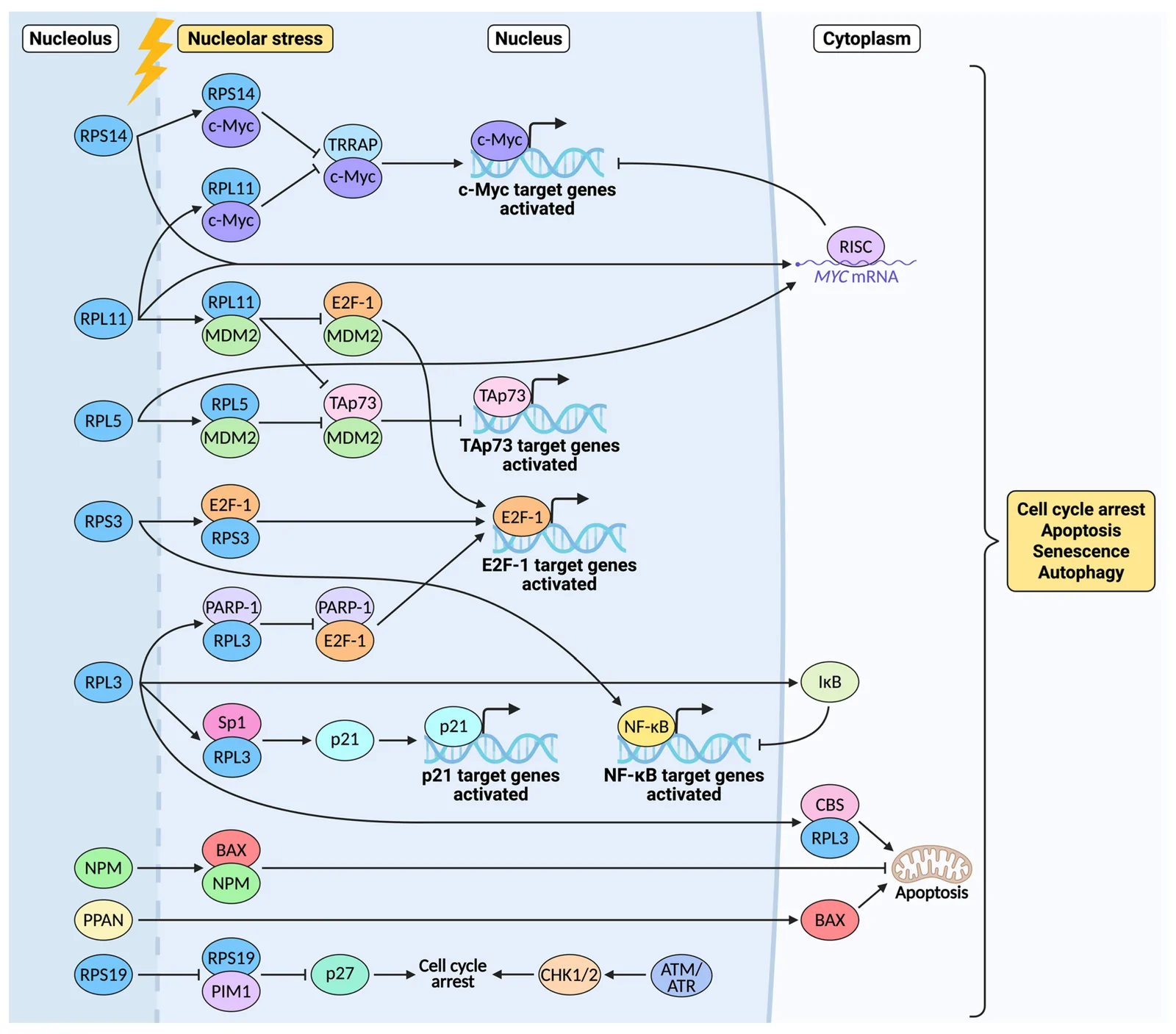
**p53-Independent Nucleolar Surveillance Pathways.** Nucleolar stress activates a diverse network of p53-independent responses that regulate cell cycle arrest, apoptosis, senescence, and/or autophagy. These pathways involve ribosomal, nucleolar, and nuclear proteins that modulate c-Myc, TAp73, E2F-1, p21, and NF-κB transcriptional programs, as well as mitochondrial apoptotic pathways and cell cycle control, operating differentially across distinct stressors and cellular contexts.

While the p53-dependent NSP is the most extensively described pathway, p53-independent mechanisms have also been reported, involving diverse effector proteins and signalling routes. These are summarised below and in Figure 6.

**E2F-1 regulation:** In p53-null cells, inhibition of rRNA synthesis by RNA polymerase I subunit A (*POLR1A*) knockdown reduces E2F transcription factor 1 (E2F-1) levels through RPL11-pRb-dependent sequestration of MDM2, leading to Skp1-Cullin-1-F-box (SCF) protein containing Skp2 complex (SCF^Skp2^)-mediated E2F-1 degradation and G1 arrest [201]. RPL3 similarly induces arrest by disrupting PARP1, E2F-1 interactions and repressing cyclin D1 expression, while also exerting pro-apoptotic and anti-autophagy functions [202,203,204,205]. RPS3 can also induce apoptosis through E2F-1, an effect suppressed by AKT-mediated phosphorylation of RPS3, which disrupts the RPS3-E2F-1 interaction [206].

**p21 induction and mitochondrial regulation:** RPL3 activates ERK/specificity protein 1 (Sp1) signalling to upregulate p21 in p53-null cells and represses cystathionine-β-synthase (CBS) expression to promote mitochondrial apoptosis under fluorouracil (5-FU) stress [205,207,208,209].

**NF-κB pathway:** Nucleolar disruption activates NF-κB through CDK4 inhibition and RRN3 degradation [210,211]. RPL3 stabilises IκBα, suppressing NF-κB signalling, while phospho-RPS3 enhances NF-κB pro-survival activity [212,213].

**RP-mediated regulation of c-Myc:** Multiple RPs regulate c-Myc expression to control cell growth. RPL11 and RPS14 bind c-Myc protein to block TRRAP recruitment and promote *MYC* mRNA decay through recruitment of the miRISC complex [214,215]. RPL5 and RPL11 also cooperate with RISC to suppress *MYC* mRNA expression [216].

**PIM1 destabilisation and cell cycle arrest:** PIM1 destabilises under ribosomal stress, which reduces p27 phosphorylation and induces cell cycle arrest in p53-null cells [197].

**BAX-mediated apoptosis:** In cells lacking p53, NPM relocalises to sequester BCL2-associated X protein (BAX) and suppress apoptosis [217]. Peter Pan homolog (PPAN), required for rRNA maturation, undergoes caspase-dependent degradation following stress, destabilising NPM and UBF while activating BAX-mediated apoptosis [218].

**CHK1/2 and ATM/ATR activation and cell cycle arrest:** In p53-null models, Pol I inhibition by CX-5461 activates CHK1/2 and G2/M arrest [219]. Furthermore, alkylating agents upregulate p14^ARF^ and trigger ATM/ATR activation, causing G2 arrest [220].

**TAp73 stabilisation:** RPL5 and RPL11 also interact with p53 homolog TAp73 to promote S phase arrest and apoptosis independently of p53 and MDM2 [221].

Collectively, these studies demonstrate that nucleolar stress responses are not confined to the canonical MDM2-p53 axis but instead involve a broad network of stress effectors that enable p53-independent surveillance. However, how these pathways are integrated and their relative contributions across different cellular and stress contexts remain unclear.

## 6. The Nucleolus in Disease

Given its central roles in RiBi and coordination of cellular stress responses, the nucleolus is essential for maintaining cellular homeostasis. Consequently, perturbations to nucleolar function can profoundly disrupt cellular physiology and contribute to pathological outcomes [222]. The relationship between the nucleolus and disease has been recognised by pathologists for over a century, with altered nucleolar morphology and number serving as disease biomarkers [223]. Reflecting its broad functional scope, nucleolar dysfunction is implicated in diverse diseases, including cancer, ribosomopathies, premature ageing, neurodegeneration, immune dysfunction, and viral infection [222]. The following sections discuss the roles of the nucleolus across these disease contexts.

### 6.1. Cancer

#### 6.1.1. Nucleolar Hypertrophy and Elevated Ribosome Biogenesis

Aberrant increases in nucleolar number and size, reflecting elevated RiBi, have been recognised as a cancer hallmark since before the 1900s and correlate with poor prognosis across tumour types [223,224,225]. The importance of elevated RiBi to tumour growth is well established, with early evidence from an Eμ-Myc-driven Burkitt lymphoma mouse model demonstrating that excessive Pol I-mediated rDNA transcription is a critical driver of malignant transformation [226,227,228]. Accordingly, hyperactivated RiBi constitutes a core feature of sustained proliferative signalling in cancer [229].

#### 6.1.2. Pathways Driving Hyperactivated Ribosome Biogenesis

In cancer cells, hyperactivated RiBi primarily arises from disruption of tumour suppressors, such as PTEN, pRb, and p53, and activation of oncogenic pathways, including PI3K/AKT/mTOR, RAS/RAF/ERK, and c-Myc (Figure 3) [42,63,230,231,232,233]. These converge to upregulate Pol I transcription by increasing expression of Pol I subunits and transcription factors such as UBF and RRN3, and in some contexts, by activating previously silent rDNA repeats [227,234]. Consistent with this, *PTEN* loss or *PIK3CA* activation occurs in about one-third of sporadic tumours [235,236], *RAF* and *RAS* are mutated in ~6–7% and ~30% of cancers, respectively [237,238], and *MYC* is amplified or dysregulated in over 70% of malignancies [63,239].

#### 6.1.3. Ribosomal Protein Dysregulation in Cancer

Consistent with elevated RiBi, overexpression of specific RPs correlates with poor prognosis across multiple cancers, including RPL35 and RPL15 in breast cancer, RPS20 and RPS11 in glioblastoma, and RPLP2, RPLP1 and RPLP0 in ovarian cancer [240,241,242]. Conversely, somatic mutations or deletions occur in several RP genes across diverse sporadic cancers, highlighting a complex and context-dependent role for RPs in tumorigenesis [243,244,245,246,247,248,249,250]. Extra-ribosomal functions of RPs may further contribute to oncogenesis; for example, loss of RPs that repress c-Myc expression, such as RPS14, RPL5, and RPL11, may promote malignant transformation, as demonstrated in lymphoma models with *Rpl11* haploinsufficiency [215,216,250,251,252].

#### 6.1.4. Evasion of the Nucleolar Surveillance Pathway

Malignant cells are frequently exposed to stressors that activate NSP responses, suggesting that suppression of NSP-induced p53 activation may confer a selective advantage during malignant transformation [32,253]. Consistent with this, *TP53* is the most frequently mutated gene in cancer, with alterations present in ~50% of tumours [254]; while this enables evasion of p53-dependent NSP responses, p53-independent NSP pathways may remain active. Supporting a model of NSP-dependent selection pressure, heterozygous deletions or inactivating mutations in RP genes occur in ~43% of human cancers, almost exclusively in tumours harbouring *TP53* mutations, and are underrepresented in *TP53* wild-type cancers [243,255]. These observations suggest that sustained NSP activation triggered by RP depletion is incompatible with malignant transformation unless p53 signalling is inactivated [32,255].

### 6.2. Ribosomopathies

Ribosomopathies are a group of largely developmental disorders caused by genetic abnormalities that impair RiBi and function (detailed in Table 1) [256,257]. These disorders arise from disruptions at multiple stages of RiBi, including rDNA transcription, rRNA processing, and/or ribosomal subunit assembly.

Diamond–Blackfan Anaemia Syndrome (DBAS), a ribosomopathy first described almost a century ago, is predominantly caused by RP haploinsufficiency, most commonly in *RPS19* (~25% of cases), with biallelic variants in *HEATR3* recently identified as an additional causative lesion [33,258,259]. Several other congenital syndromes have since been linked to impaired RiBi, including Shwachman–Diamond Syndrome (SDS), primarily caused by loss-of-function mutations in the 60S maturation factor *SBDS* [260]; myelodysplastic neoplasm with low blast and isolated del(5q) (MDS-5q), resulting from deletion of the long arm of chromosome 5, encompassing 40 genes including *RPS14*, *CSNK1A1*, *EGR1*, *HSPA8*, miR-145, and miR-146a [261]; Treacher Collins Syndrome (TCS), caused by mutations in the rRNA transcription and processing factor *TCOF1* or in Pol I subunits *POLR1B*, *POLR1C*, or *POLR1D* [262]; X-linked Dyskeratosis Congenita (DC), resulting from variants in *DKC1*, which catalyses rRNA pseudouridylation [263]; Bowen–Conradi Syndrome (BCS), caused by mutations in the RNA methyltransferase and 40S biogenesis factor *EMGI* [264]; Cartilage-Hair Hypoplasia (CHH), caused by mutations in *RMRP*, which functions in pre-rRNA processing [265]; North American Indian childhood cirrhosis (NAIC), resulting from mutations in *UTP4/CIRH1A*, a pre-rRNA processing factor [266]; and acrofacial dysostosis, Cincinnati type, caused by loss-of-function mutations in the Pol I subunit *POLR1A* [267].

Additionally, less common ribosomopathies have been linked to mutations in RP genes and RiBi factors (e.g., *RPSA*, *RPS23*, *RPL10*, *RPL13*, *RPL21*, *AIRIM, RBM28*, *BMSI,* and *TAF1A*), further emphasising that disruption of diverse components of the ribosome production pathway can converge on pathological phenotypes [268,269,270,271,272,273,274,275,276,277,278,279,280,281,282,283].

**Table 1:** **Genetic, Molecular, and Clinical Features of Inherited and Acquired Ribosomopathies.**

Ribosomopathy	Genetic Lesion (Frequency)	Inheritance (Where Relevant)	Function in Ribosome Biogenesis	Cancer Susceptibility	Clinical Features	Current Treatment	Incidence	p53 Implicated	References
Diamond–Blackfan Anaemia Syndrome (DBAS)	*RPS19* (~25%), *RPL5* (7–10%), *RPL11* (~5%), *RPS26* (3–6%), *RPS10* (3–6%), *RPL35A* (2–4%), *RPS24* (2–4%), *RPS17* (1–3%), *RPS7, RPS15A, RPS20, RPS27*, *RPS28*, *RPS29*, *RPL4*, *RPL8*, *RPL9*, *RPL15, RPL17*, *RPL18*, *RPL26*, *RPL27*, *RPL31*, *RPL35, TSR2, HEATR3, GATA1, TP53* (GOF) (<1%)	*RPs* (autosomal dominant), *TSR2* and *GATA1* (X-linked), *HEATR3* (autosomal recessive), *TP53* (GOF)	Ribosomal subunit assembly, rRNA processing	Absent/reduced and left-shifted erythroid precursors, severe macrocytic anaemia, reticulocytopenia, short stature, colitis, congenital abnormalities (craniofacial and neck (commonly cleft lip and/or palate), thumb and skeletal, cardiac, urogenital, neurodevelopmental, ophthalmological), immune system abnormalities (lymphocytes and serum immunoglobulin defects)	AML, MDS, osteosarcoma, colon, non-Hodgkin lymphoma, thyroid cancer, gastric cancer, breast cancer, multiple myeloma, cardiac Purkinje cell tumour, DLBCL, oesophageal cancer, vaginal squamous cell carcinoma	Corticosteroids, RBC transfusions, HSCT, L-leucine	~5–10 case per million live births	Yes	[259,284,285,286]
Shwachman–Diamond Syndrome (SDS)	*SBDS* (90%), *EFL1*, *DNAJC21*, *SRP54*	Autosomal recessive	Ribosomal subunit assembly, rRNA processing	Exocrine pancreatic insufficiency, neutropenia or multilineage cytopenias, thrombocytopenia, skeletal abnormalities (osteopenia, rib cage dysplasia, chondrodysplasia, metaphyseal), eczema, attention deficit disorder, transient hepatitis, failure to thrive	MDS, AML, lymphoma, ovarian cancer	RBC transfusions, pancreatic enzyme supplementation, G-CSF, vitamin supplements, HSCT, reconstructive surgery	~1 case per 168,000 live births	Yes	[260,287,288]
Myelodysplastic neoplasm with low blast and isolated del(5q) (MDS-5q)	Deletion of 40 genes in 5q31 (CDR1) and 5q32-5q33 (CDR2), including *RPS14*, *RPL7*, *RPLP1*, *CSNK1A1*, *EGR1*, *HSPA8*, *SPARC*, *LARP1*, miR-145, miR-146a	- (Somatic heterozygous deletion)	Ribosomal assembly, rRNA processing	Erythroid hypoplasia, macrocytic anaemia, elevated/normal platelet count, hypolobulated megakaryocytes with <5% bone marrow myeloblasts, <1% circulating peripheral blasts, thrombocytosis	10% progression to AML	RBC transfusions, erythropoietin, hypomethylating agents, chemotherapy, thalidomide, retinoids, lenalidomide, HSCT	~1 case per million live births	Yes	[261,289,290,291]
Treacher Collins Syndrome (TCS)	*TCOF1* (86%), *POLR1D* (6%), *POLR1C* (1.2%), *POLR1B* (1.3%)	*TCOF1*, *POLR1D*, and *POLR1B* (autosomal dominant), *POLR1D* and *POLR1C* (autosomal recessive)	rRNA transcription and processing	Bilateral craniofacial abnormalities (mandibular and malar hypoplasia, microtia accompanied by conductive hearing loss, cleft lip, cleft palate, lower lid coloboma, downward-slanting palpebral fissures), extra-craniofacial anomalies (limb, CNS, reproductive, cardiac, vertebral), intellectual disability, microcephaly	None reported	Reconstructive surgery	~1 in 50,000 live births	Yes	[262,292,293]
X-linked Dyskeratosis Congenita (DC)	*DKC1* (25% of DC cases)	X-linked	rRNA processing	Mucosal leukoplakia, nail dystrophy, skin hyperpigmentation, oral mucosa, pulmonary fibrosis, premature ageing, anaemia, thrombocytopenia	MDS, AML, head and neck squamous cell carcinomas, skin cancer, anorectal cancer, tongue cancer	RBC transfusions, androgens (oxymetholone), HSCT	~1 case per 4 million live births	Yes	[263,294,295,296]
Cartilage-Hair Hypoplasia (CHH)	*RMRP*	Autosomal recessive	rRNA processing	Anaemia, immunodeficiency affecting T-cell response, bone deformities, short-limbed dwarfism, fine and sparse hair, gastrointestinal malabsorption, neutropenia	Non-Hodgkin lymphoma, basal cell carcinoma	RBC transfusion, G-CSF, HSCT, reconstructive surgery	~1 case per 23,000 live births	No	[265,297,298,299]
Bowen–Conradi Syndrome (BCS)	*EMG1*	Autosomal recessive	Ribosomal subunit assembly, rRNA processing	Impaired postnatal and prenatal growth, psychomotor delay, rocker-bottom feet, camptodactyly, joint malformations (flexion contractures), micrognathia, prominent nose with absent glabellar angle, microcephaly, CNS defects, bone marrow failure, early infant death	Not determined	None	~1 case per 355 live births within the Hutterite population of Canada and the United States	No	[264,300,301,302]
North American Indian childhood cirrhosis (NAIC)	*UTP4/CIRH1A*	Autosomal recessive	rRNA processing	Transient cholestatic neonatal jaundice, hepatosplenomegaly, biliary cirrhosis, portal hypertension	Not determined	Liver transplantation	~30 cases reported in the Cree-Ojibway First Nation population of Northern Quebec	Yes	[266,303,304,305]
Acrofacial dysostosis, Cincinnati type	*POLR1A*	Autosomal dominant	rRNA transcription	Limb defects, craniofacial anomalies, structural cardiac defects, neurodevelopmental abnormalities	Not determined	NA	~20 cases reported	Yes	[267,306,307]
RPSA-associated Isolated Congenital Asplenia (ICA)	*RPSA* (~40% of ICA cases)	Autosomal dominant	Ribosomal subunit assembly, rRNA processing	Absence of a spleen	Not determined	Anti-infection and antibiotic prophylaxis, vaccination (influenza, meningococcal, haemophilus influenzae type b, Pneumococcal)	~1 case per 5 million births	No	[308,309]
RPL13-associated spondyloepimetaphyseal dysplasia (SEMD-RPL13)	*RPL13*	Autosomal dominant	Ribosomal subunit assembly, rRNA processing	Severe skeletal and bone dysplasia, short statue	Not determined	Lower extremity surgery, growth hormone	~27 cases reported	No	[268,269,270,271,272]
AIRIM ribosomopathy	*AIRIM/C1orf109*	Autosomal recessive	Ribosomal subunit assembly	Developmental delay, intellectual disability, microcephaly, muscular hypotonia accompanied by limb spasticity and dystonia, vision and hearing impairment, dysmorphism, infantile seizures	Not determined	NA	~18 cases reported	No	[283]
RPL10 ribosomopathy	*RPL10*	X-linked	Ribosomal subunit assembly	Microcephaly, growth retardation, seizures, craniofacial defects, genitourinary abnormalities, hand and food malformations, intellectual disability	Not determined	NA	~15 cases reported	No	[276,277,278,279]
BMS1-associated Aplasia Cutis Congenita (ACC)	*BMS1*	Autosomal dominant	Ribosomal subunit assembly, rRNA processing	Congenital localised absence of skin, mostly affecting the scalp vertex	Not determined	Antibiotics, surgical repair, skin grafting	~11 cases reported	p53-independent increase in p21 levels	[273]
Alopecia, Neurologic Defects, and Endocrinopathy (ANE) Syndrome	*RBM28*	Autosomal recessive	Ribosomal subunit assembly, rRNA processing	Alopecia, hair and skin abnormalities, neurological defects, intellectual disability, motor deterioration, craniofacial malformations, endocrinopathy, hypoplastic pituitary, hormonal deficiencies with pubertal delay	Not determined	NA	~6 cases reported	No	[274,310,311,312]
RPL21-associated Hereditary Hypotrichosis Simplex (HHS)	*RPL21*	Autosomal dominant	Ribosomal subunit assembly	Hair loss affecting scalp, body, eyebrows, eyelashes	Not determined	NA	~4 cases reported	No	[275,313]
TAF1A ribosomopathy	*TAF1A*	Autosomal recessive	rRNA transcription	End-stage dilated cardiomyopathy	Not determined	Heart transplantation	~3 cases reported	No	[281]
RPS23 ribosomopathy	*RPS23*	Unknown	Ribosomal subunit assembly	Brachycephaly, trichomegaly, developmental delay, microcephaly, hearing loss, dysmorphic features	Not determined	NA	~2 cases reported	No	[280]

**Key**: RP = ribosomal protein; GOF = gain-of-function; AML = acute myeloid leukaemia; MDS = myelodysplastic syndrome; DLBCL = diffuse large B-cell lymphoma; CDR = commonly deleted region; CNS = central nervous system; RBC = red blood cell; HSCT = haematopoietic stem cell transplantation; G-CSF = granulocyte colony-stimulating factor; NA = not available.

#### 6.2.1. Clinical Features and Pathogenic Mechanisms of Ribosomopathies

Despite arising from ubiquitous defects in RiBi, ribosomopathies manifest with striking tissue specificity, most commonly involving bone marrow failure and craniofacial or skeletal abnormalities (Table 1) [256]. How disruption of such a fundamental cellular process produces these selective phenotypes remains a central and unresolved question. Notably, ribosomopathies presenting with paediatric bone marrow failure, including DBAS, SDS, MDS-5q, X-linked DC, and CHH, are associated with a significantly increased risk of malignancy later in life, with overall cancer incidence estimated at 2.5- to 8.5-fold higher than in the general population and up to 200-fold for specific cancer types [284,297,314,315,316,317,318]. Below, we discuss current models of ribosomopathy pathogenesis and proposed mechanisms linking ribosome dysfunction to tissue-specific phenotypes and cancer susceptibility.

##### Mechanisms Underlying Tissue Specificity in Ribosomopathies

Several mechanisms have been proposed to explain the tissue-specific phenotypes observed in ribosomopathies, as outlined below.

**Affected cell lineages and proliferative demand:** A consistent feature of multiple ribosomopathies is the selective vulnerability of neural crest (NC)-derived tissues, resulting in craniofacial defects, intellectual disability, cardiac abnormalities, skin and pigmentation defects, and hearing loss (Table 1) [259,260,262,263]. Ribosome levels decline during human neuroepithelial differentiation, although protein synthesis must be maintained above a minimum threshold to support cell fate specification and survival [283]. Differentiating cells may therefore be particularly vulnerable to perturbations in RiBi, with failure to meet this threshold during critical developmental windows potentially contributing to these neurodevelopmental phenotypes [283].

The haematopoietic system is also frequently affected (Table 1), commonly presenting as bone marrow failure, likely reflecting the high proliferative demand and reliance on elevated protein synthesis in these cells [259,260,262]. However, the relative sparing of other highly proliferative tissues, such as the intestine, indicates that proliferative demand alone does not fully explain tissue specificity.

**p53-driven pathogenesis:** The hypo-proliferative phenotypes in ribosomopathies are thought to arise, at least in part, from aberrant activation of the p53-dependent NSP in response to impaired RiBi. Multiple animal and patient studies implicate p53 activation in disease phenotypes across several ribosomopathies (Table 1), with partial phenotypic rescue observed upon p53 depletion [306,319,320,321,322,323,324,325]. Tissue specificity may reflect cell type-specific differences in p53 sensitivity. For example, NC progenitors exhibit heightened p53 sensitivity within defined developmental windows [137,326], and erythroid progenitors appear disproportionately sensitive to p53 stabilisation [321,322,324,327]. The mechanisms underlying this lineage-specific sensitivity remain incompletely understood. p53 also regulates erythroid progenitor and immature precursor proliferation [328]; therefore, in ribosomopathies with impaired erythropoiesis, aberrant activation may impair erythroblast expansion both by enhancing its anti-proliferative effects and by disrupting this normal regulatory role. Nevertheless, not all ribosomopathy phenotypes are p53-dependent; for example, biallelic *HEATR3* variants cause DBAS through a p53-independent mechanism [33], indicating that p53-dependent NSP activation is a major but not exclusive driver of pathogenesis.

**Oxidative stress:** Oxidative stress has emerged as an additional contributor, with elevated ROS reported in DBAS, DC, and SDS [329,330,331,332]. In DBAS, impaired RiBi slows globin synthesis while heme production proceeds at a relatively normal rate, generating excess free heme that promotes ROS production, lipid peroxidation, and apoptosis in erythroid cells [329,333]. Distinct mechanisms also contribute in other contexts. For example, in SDS, p53 activation in niche mesenchymal cells triggers secretion of inflammatory mediators that induce oxidative stress in neighbouring haematopoietic stem and progenitor cells (HSPCs) [334], while in TCS, impaired DNA damage repair in ROS-sensitive neuroepithelial and NC cells drives apoptosis and craniofacial pathology [131,136,335].

**Translational dysregulation:** Translational dysregulation may also contribute through two models. The specialised ribosome hypothesis proposes that RP defects alter ribosome composition and confer selective translational capacity [336,337], whereas the ribosome concentration hypothesis proposes that reduced ribosome availability selectively impairs translation of specific mRNAs [338]. In DBAS, impaired translation of *GATA1* mRNA illustrates the latter mechanism [338,339,340]. Similarly, variants in the 60S biogenesis factor *AIRIM* reduce ribosome availability and disproportionately impair translation of specific transcripts, disrupting neuroepithelial fate commitment and survival, with these phenotypes partially rescued by enhancing mTOR signalling to increase global protein synthesis [283]. Additionally, these mechanisms likely intersect with p53-dependent responses, including p53-mediated suppression of cap-dependent translation via 4E-BP1 [341].

Collectively, these mechanisms illustrate how disrupted RiBi can alter cellular state in a highly context-dependent manner, giving rise to tissue-specific phenotypes. However, the relative contributions of these mechanisms, their potential interplay, and their causal roles in specific phenotypes remain key unresolved questions.

##### Cancer Susceptibility in Ribosomopathies

Several mechanisms may explain the shift from early hypo-proliferative phenotypes to later hyper-proliferation and cancer susceptibility in ribosomopathies associated with bone marrow failure, including somatic genetic rescue (SGR) and translational dysregulation.

**Somatic genetic rescue:** SGR involves the acquisition of mutations that mitigate germline defects and has been reported in SDS, DBAS, and MDS-5q [342,343,344,345,346,347,348,349]. While SGR can restore cellular fitness, it may also enable clonal expansion and increase the risk of malignant progression [342,343]. A key SGR event is selection for *TP53* loss-of-function mutations driven by chronic p53 activation, enabling cells to evade apoptosis and cell cycle arrest [350]. This provides a proliferative advantage but removes a critical tumour suppressor barrier, thereby facilitating malignant transformation [350]. SGR can also restore RiBi defects directly; for example, in SDS, deletions reducing eIF6 dosage alleviate its inhibition of 60S maturation [342,345,351].

**Translational dysregulation:** Translational dysregulation may promote oncogenesis through “onco-ribosomes”, in which altered ribosome composition or fidelity biases translation toward oncogenic transcripts [350]. For example, *RPS15* variants in chronic lymphocytic leukaemia (CLL) reduce translational fidelity [352], and *RPL10* mutations in paediatric T-cell acute lymphoblastic leukaemia increase BCL-2 expression and JAK-STAT signalling [353].

Together, these mechanisms illustrate how initial defects in RiBi can create strong selective pressure for adaptations that restore growth while simultaneously lowering barriers to malignant transformation.

### 6.3. Other Nucleolus-Associated Pathologies

Beyond cancer and ribosomopathies, nucleolar dysfunction is implicated in premature ageing syndromes, neurodegenerative disorders, and infectious and immune conditions, as outlined below.

#### 6.3.1. Ageing and Neurodegeneration

Studies of premature ageing syndromes and neurodegenerative disorders support a link between ageing, neurodegeneration, and altered nucleolar morphology and function [354,355], consistent with longstanding hypotheses implicating rDNA instability in cellular senescence and ageing [93,356]. Premature ageing syndromes caused by defects in DNA repair, including Cockayne syndrome (CS) and Werner syndrome (WS), show reduced nucleolar activity associated with impaired rDNA transcription [357,358]. In contrast, Hutchinson–Gilford progeria syndrome exhibits enlarged nucleoli driven by increased rDNA transcription and RiBi [359]. These observations indicate that both reduced and dysregulated nucleolar activity can contribute to ageing-associated phenotypes.

Many neurodegenerative diseases display nucleolar abnormalities resembling those in CS and WS. Nucleolar disruption is observed in Parkinson’s disease (PD) [360], and *Rrn3* deletion in mouse dopaminergic neurons induces a PD-like phenotype with p53-dependent apoptosis [361]. Reduced nucleolar size, function, and translational capacity have also been reported in Alzheimer’s disease (AD) [362,363]. Heterozygous gain-of-function *UBTF* mutations are linked to childhood neuroregression syndrome [364]. Familial amyotrophic lateral sclerosis (ALS) driven by *C9ORF72* mutations displays enlarged nucleoli associated with accumulation of toxic peptides and impaired RiBi and translation, suggesting nucleolar enlargement reflects stress rather than increased RiBi [365,366,367,368]. Nucleolar dysfunction has also been implicated in Huntington’s disease (HD), where post-translational UBF modifications reduce rDNA transcription, contributing to striatal dysfunction and neurodegeneration [369,370,371].

Across these conditions, nucleolar dysfunction converges on impaired proteostasis, stress signalling, and neuronal survival, highlighting a shared mechanistic link between RiBi dysregulation and neurodegeneration.

#### 6.3.2. Infectious and Immune Disease

The nucleolus also contributes to host responses during viral and bacterial infections and may influence autoimmune disorders, as outlined below.

##### Viral Infection

Interactions between viruses and the nucleolus have been recognised for decades [110,372,373]. Viruses exploit host cellular machinery, and the nucleolus, as a key regulator of RiBi, RNA metabolism, and stress responses, is a frequent target of viral manipulation [110,372,373].

Viral proteins often localise to the nucleolus via NoLS or chaperones, interacting with nucleolar components to facilitate transcription, RNA maturation, or particle assembly [110,372]. Infection can also drive redistribution of nucleolar proteins to the nucleoplasm or cytoplasm, while recruiting non-nucleolar factors into nucleoli to support viral replication [110,372]. Viruses may further reshape host cell physiology by altering cell cycle progression, apoptosis, and RiBi [110]. These processes are accompanied by pronounced changes in nucleolar morphology and composition, reflecting functional reprogramming of the nucleolus during infection [373].

These interactions are observed across diverse RNA and DNA viruses, including *Flaviviridae* (e.g., Hepatitis C virus), *Coronaviridae* (e.g., SARS coronavirus), *Orthomyxoviridae* (e.g., Influenza A), *Retroviridae* (e.g., HIV), and *Herpesviridae* (e.g., Epstein–Barr virus), indicating that nucleolar targeting is a conserved strategy to co-opt host cellular machinery [110,222,372,373,374,375].

##### Bacterial Infection

The nucleolus also participates in host immune responses to bacterial pathogens. NCL recruits Rrp6-exosomes to the nucleolus to degrade cytosine/uracil-rich inflammatory pre-mRNAs, limiting inflammatory gene expression [111]. Accordingly, Ncl depletion in mice leads to excessive inflammatory responses and lethality following bacterial lipopolysaccharide (LPS) exposure [111]. *Staphylococcus aureus*, *Acinetobacter baumannii,* and *Legionella pneumophila* infection, also induce changes in nucleolar number and morphology [109]. Together, these observations suggest that nucleolar function may undergo dynamic remodelling during host–pathogen interactions.

##### Autoimmune Disorders

The involvement of the nucleolus in autoimmune disease is an emerging area, with mechanistic links yet to be fully established. Dysregulated microRNA expression is a feature of multiple autoimmune disorders, including Sjögren’s syndrome, rheumatoid arthritis, systemic lupus erythematosus (SLE), Hashimoto’s thyroiditis, and Graves’ disease, raising the possibility that microRNA-associated nucleolar pathways may contribute to autoimmune dysregulation, although a direct causal role has not been established [222,376]. Similarly speculative is the “X chromosome-nucleolus nexus”, which proposes that stress-induced nucleolar expansion may engulf and disrupt adjacent chromatin regions, with the inactive X chromosome (Xi) potentially being especially susceptible because of its close proximity to the nucleolus. This may destabilise Xi, leading to aberrant expression of normally silenced X-linked genes and Xi-associated *Alu* elements, which could trigger immune responses and contribute to the female bias observed in autoimmune disorders [377,378,379]. Additionally, nucleolar proteins, including NCL and FBL, possess autoantigenic properties, and their release during cell death may stimulate production of antinuclear autoantibodies (ANAs) [380]. While these observations collectively hint at a role for nucleolar dysfunction in immune dysregulation, the underlying mechanisms require further experimental validation.

Collectively, these findings establish the nucleolus as a functionally important contributor to a broad spectrum of human diseases and disorders, encompassing cancer, ribosomopathies, ageing, neurodegeneration, and immune conditions. The recurrence of nucleolar dysfunction across such diverse pathological contexts presents opportunities for therapeutic exploitation.

## 7. Therapeutically Targeting the Nucleolus in Disease

The preceding sections highlight the central role of nucleolar dysfunction in human pathologies, prompting interest in therapeutically targeting key nucleolar processes, particularly RiBi and the NSP. A variety of mechanisms have been explored to directly modulate nucleolar function, including agents that influence RNA polymerase transcription (such as Pol I destabilisers, Pol I elongation inhibitors, and Pol III and rRNA processing modulators), ribosome assembly, nucleolar structure, and signalling pathways closely linked to RiBi. These mechanisms have been recently reviewed [381,382], and while some will be discussed below in the context of specific disease settings, others will not be addressed in detail.

Cancer and ribosomopathies illustrate opposite extremes of nucleolar dysregulation. In many cancers, hyperactivated RiBi drives proliferation, whereas ribosomopathies feature impaired RiBi and, frequently, excessive p53-dependent NSP activation. Consequently, therapeutic strategies differ: in cancer, interventions aim to suppress RiBi and leverage NSP activity to limit proliferation or induce cancer cell apoptosis, while in ribosomopathies, treatments focus on mitigating downstream consequences, such as bone marrow failure, or restoring RiBi through DNA- or RNA-based genetic approaches. These strategies are discussed in the following sections.

### 7.1. Therapeutic Targeting of the Nucleolus in Cancer

#### 7.1.1. Small-Molecule Inhibitors Targeting Pol I Activity

The established role of hyperactivated RiBi in malignant transformation has driven the development of agents designed to selectively inhibit this process [382]. These include small-molecule therapies directly targeting Pol I activity, including CX-3543 [383,384], BMH-21 [385,386], and CX-5461 (Pidnarulex) [227,387,388,389]. CX-3543 binds rDNA G-quadruplex (G4) structures and disrupts their interaction with NCL, thereby inhibiting Pol I activity [383]. BMH-21 intercalates into G4 and GC-rich regions, which are particularly abundant in rDNA, and promotes degradation of the Pol I subunit RPA194 [385,386]. CX-5461 has emerged as a leading agent and is discussed below.

#### 7.1.2. CX-5461: Preclinical Activity, Clinical Evaluation, and Mechanism of Action

**Preclinical Activity:** Preclinical studies have demonstrated robust anti-cancer activity of CX-5461. In Acute Myeloid Leukaemia (AML) and lymphoma, CX-5461 selectively eliminates malignant cells via activation of the p53-dependent NSP and subsequent cell cycle arrest and apoptosis, where sensitivity correlated with p53 status and RiBi activity [219,227]. CX-5461 extends survival in p53-null AML models through late S and G2/M arrest/delay mediated by ATM/ATR and CHK1/CHK2 signalling [227]. Anti-cancer activity has been demonstrated across a broad range of solid and haematological malignancies, consistent with a general dependency on elevated RiBi in cancer cells [387,390,391,392,393,394].

**Clinical Evaluation:** Clinical evaluation of CX-5461 has confirmed its therapeutic potential. A completed Phase I trial in advanced haematologic malignancies and breast cancer demonstrated activity (NCT02719977) [389]. Ongoing Phase Ib studies are assessing tolerability in solid tumours, including pancreatic, ovarian, and breast cancers (NCT04890613). CX-5461 has received FDA fast-track designation for the treatment of DNA damage repair-deficient cancers [395].

**Mechanism of Action:** CX-5461 is a quinazolinone-derived small-molecule inhibitor of Pol I transcription, identified through functional screening for selective suppression of rDNA transcription [387]. It exhibits ~200-fold greater potency against Pol I relative to Pol II, acting primarily by disrupting SL1-dependent PIC formation at rDNA promoters, thereby blocking Pol I transcription initiation without directly affecting Pol II activity or global transcription [219,227,387]. Subsequent studies identified additional mechanisms, including G4 stabilisation, nucleolar R-loop formation, promoter escape inhibition, and topoisomerase II inhibition [391,392,396,397,398,399,400]. Topoisomerase II inhibition provides a plausible basis for the DNA damage signalling observed in some cellular contexts, consistent with ATM/ATR pathway activation [397,398,399,400]. However, the relative contribution of these mechanisms to its anti-cancer effects remains unresolved. Nevertheless, nucleolar stress signalling induced by Pol I transcription inhibition is likely the dominant driver of therapeutic efficacy [219,227].

**Mutagenicity:** Concerns regarding mutagenicity have been raised in *in vitro* studies reporting non-selective mutagenesis at levels exceeding environmental carcinogens [400]. However, these findings have not been reproduced across independent experimental systems, and clinical sequencing data in a Phase I trial showed no significant increase in mutational burden (ACTRN12613001061729). These observations indicate that clinical activity is not primarily driven by genotoxic mechanisms, and that the *in vitro* mutagenic effects likely reflect context-specific or off-target effects rather than an inherent property of Pol I inhibition.

#### 7.1.3. Next-Generation Pol I Inhibitors: PMR-116

To address concerns around DNA damage and to enable improved therapeutic index and potential application in settings such as central nervous system malignancies, next-generation selective Pol I inhibitors with reduced off-target activation of DNA damage signalling have been developed, including PMR-116 [401]. PMR-116 arrests Pol I at the rDNA promoter, preventing promoter escape and reducing elongation, representing a more selective suppression of RiBi compared to the multi-mechanistic activity described for CX-5461 [401]. In preclinical models, it activates the p53-dependent NSP, delays cell cycle progression, and suppresses E2F and c-Myc signalling, with *MYC*-driven cancers being particularly sensitive [401]. Importantly, it does not induce CHK1/2 phosphorylation or global γH2AX foci formation, suggesting that PMR-116 mediates its anti-cancer effects independent of DNA damage [401]. Early Phase I data show on-target activity, with ~50% reduction in rRNA synthesis in peripheral blood cells 24 h post-treatment (CTRN12620001146987), and the compound has progressed to Phase II evaluation [402].

Together, these studies highlight that selective targeting of Pol I transcription can be refined to dissociate nucleolar stress signalling from DNA damage responses, providing a path toward effective and potentially less genotoxic cancer therapies.

### 7.2. Therapeutic Strategies for Ribosomopathies

#### 7.2.1. Current Therapeutic Strategies

Bone marrow failure is the predominant cause of morbidity in both inherited and acquired ribosomopathies, arising from impaired RiBi that disrupts haematopoietic progenitor proliferation and survival [256,257]. Therapeutic strategies are primarily supportive and tailored to the specific cytopenias present, with the aim of maintaining haematopoiesis, reducing complications, and preserving organ function prior to consideration of curative therapy [403]. Importantly, most current therapies do not directly target nucleolar function or RiBi itself but instead aim to ameliorate downstream consequences of impaired RiBi, particularly cytopenias and organ dysfunction (Table 1).

**Anaemia:** In DBAS, corticosteroids such as dexamethasone and prednisone are the first-line therapy, stimulating stress erythropoiesis and expansion of early erythroid progenitors [259,404,405,406]. Approximately 80% of patients initially respond, although only ~40% maintain long-term benefit, and ~20% are entirely refractory [407,408]. Chronic or high-dose steroid use carries risks including growth delays, osteoporosis, hypertension, cataracts, and immunosuppression [406,409]. In SDS, MDS-5q, and DC, anaemia is often managed with red blood cell transfusions; however, in MDS-5q, this may be progressively replaced by treatments such as retinoid, lenalidomide, and erythropoietin [403]. In CHH, severe anaemia requiring transfusion is uncommon [403]. These strategies are informed by disease-specific differences in haematopoiesis: DBAS is predominantly erythroid-restricted, whereas SDS, MDS-5q, DC, and CHH frequently present with multilineage marrow dysfunction [403].

**Neutropenia:** Neutropenia is prominent in SDS and can also occur in CHH [260,265]. Granulocyte colony-stimulating factor (G-CSF) is used to increase neutrophil counts and prevent infections, while haematopoietic stem cell transplantation (HSCT) is used to correct immunodeficiency [403]. Recurrent infections may exacerbate organ dysfunction, highlighting the need for early intervention [410].

**Platelet Abnormalities:** Platelet transfusions are indicated in severe thrombocytopenia, which can occur in SDS, DC, and CHH, while thrombocytosis can arise in MDS-5q, often alongside other cytopenias [265,295,410,411]. Accordingly, careful monitoring of platelet levels and related complications may be required in affected patients.

**Multilineage Therapies:** Supportive care in SDS often extends beyond marrow failure to address pancreatic insufficiency and nutritional deficiencies [403,412]. In MDS-5q, lenalidomide remains a well-established immunomodulatory therapy that can reduce transfusion dependence in many patients [261]. DBAS, SDS, TCS, and CHH patients may require management of craniofacial and skeletal abnormalities through reconstructive surgery [403].

While these interventions can partially correct cytopenias and improve quality of life, they do not address the underlying defect in RiBi or nucleolar function and are associated with long-term complications such as iron overload, endocrine dysfunction, infection, and cardiovascular disease [403,409,413]. This highlights a major therapeutic gap and the need for approaches that directly target nucleolar pathways.

#### 7.2.2. Emerging Pharmacological Therapies

Several pharmacological agents targeting disease-relevant pathways are under clinical investigation or showing translational promise across the spectrum of ribosomopathies, particularly in DBAS, SDS, and DC. However, most emerging agents similarly act indirectly, targeting stress response or translational control pathways (e.g., p53 or mTOR signalling) rather than restoring nucleolar function *per se*.

**Diamond–Blackfan Anaemia Syndrome:** In DBAS, L-leucine, an mTOR activator, has been evaluated in early clinical studies and compassionate use settings, enhancing translation and erythropoiesis with improvements in haemoglobin and growth parameters without major adverse events [259,414,415,416]. Agents that modulate p53 signalling remain of interest; the FDA-approved calmodulin inhibitor trifluoperazine reduces p53 target activation and ameliorates anaemia in preclinical DBAS models (NCT03966053) [417], prompting the development of next-generation compounds with improved safety [418]. The thrombopoietin receptor agonist eltrombopag has been evaluated to reduce toxic free heme arising from heme-globin imbalance in DBAS, showing modest haemoglobin improvement but causing dose-limiting thrombocytosis in some patients (NCT04269889) [419,420].

**Shwachman–Diamond Syndrome:** Given the high prevalence of nonsense mutations in *SBDS*, Ataluren, a small-molecule that promotes ribosomal read-through of premature stop codons, was administered to three SDS patients carrying nonsense mutations under a compassionate use program [421]. Treatment restored SBDS protein function and alleviated both pancreatic and haematopoietic dysfunction, supporting nonsense suppression as a potential therapeutic strategy [421].

**Dyskeratosis Congenita:** For DC, androgen derivatives such as danazol and oxymetholone have demonstrated haematological improvement in clinical studies, increasing haemoglobin, granulocyte, and platelet levels, and are among the few pharmacological agents with broad lineage effects [422,423]. Given the defective telomere maintenance in DC, a telomere elongation gene therapy (EXG34217) delivering Zinc finger and SCAN domain-containing 4 (ZSCAN4), a recombination-mediated telomere elongation factor, into mobilised CD34^+^ cells for autologous HSCT is under early clinical investigation (NCT04211714) [424].

Future efforts may focus on developing small molecules that target ribosomal dysfunction (e.g., in the context of DBAS, modulators of RiBi, p53 activation, and proliferation in erythroid progenitors and precursors with the aim of rescuing erythropoiesis [425,426]) to improve treatment efficacy and reduce relapse. Collectively, these pharmacological strategies illustrate a growing pipeline of small-molecule and mechanism-targeted agents that move beyond symptom management toward pathway-directed intervention, although most remain in early clinical evaluation or preclinical development.

#### 7.2.3. Allogeneic Haematopoietic Stem Cell Transplantation

Allogeneic HSCT remains the only potentially curative therapy for bone marrow failure in DBAS, SDS, MDS-5q, DC, and CHH [427,428,429,430,431]. HSCT restores multilineage haematopoiesis and can mitigate long-term complications of chronic supportive therapy [427,428,429,430,431]. Despite its curative potential, HSCT is associated with substantial morbidity and mortality, largely from infections and graft-versus-host disease (GVHD), and requires lifelong immunosuppression therapy [428,429,430,431,432]. Other complications include thrombotic microangiopathy, veno-occlusive disease, acute pancreatitis, EBV-associated lymphoproliferative disease, and secondary malignancies [428,429,430,431,432].

Ongoing clinical trials aim to improve HSCT safety and efficacy in ribosomopathies and related disorders through optimised conditioning and T- and B-cell depletion, including trials NCT04965597 (DBAS, SDS, other BMFS, haematologic neoplasms), NCT02928991 (DBAS, DC, SDS, other BMFS), NCT03513328 (DBAS, other BMFS, haemoglobinopathies, metabolic disorders, primary immunodeficiencies), NCT04099966 (DBAS, MDS, other BMFS, haematologic neoplasms, sickle cell disease), and NCT03653338 (DBAS, β-thalassemia, sickle cell disease). Nonetheless, the limitations of current HSCT regimes underscore the need for alternative approaches targeting the molecular lesions, with the goal of reducing toxicity, enhancing efficacy, and preventing secondary complications such as cancer.

### 7.3. DNA and RNA Therapeutic Approaches in Ribosomopathies and Cancer

Emerging DNA- and RNA-based therapies provide a framework for directly modulating nucleolar function and RiBi, with the potential to move beyond symptomatic management and address underlying molecular defects. These approaches may enable restoration of defective RP expression, tuning of nucleolar stress pathways, and selective targeting of nucleolar dependencies in cancer. While clinical data remain limited, early preclinical studies highlight their potential as mechanism-directed interventions. The following sections summarise current strategies and outline key challenges for their translation.

#### 7.3.1. DNA-Based Therapeutic Strategies

DNA-based approaches aim for permanent or semi-permanent correction of pathogenic variants or haematopoietic regulators. This approach has been successfully utilised across a broad range of disease conditions [433], and in ribosomopathies, *ex vivo* lentiviral delivery of wild-type *RPS19* has shown potential in rescuing erythropoiesis in *RPS19*-deficient human HSPCs and mice [434,435,436,437]. Similarly, a lentiviral construct driving erythroid-specific *GATA1* expression enhanced erythropoiesis in patient-derived HSPCs across diverse genotypes [438], reflecting observations that *GATA1* mRNA levels are reduced across multiple DBAS genotypes [339,340,438]. CRISPR-based editing, including base and prime editors, offers correction of point mutations or small insertions or deletions while maintaining endogenous gene regulation and has shown promise in correcting *RPS19* mutations in HSPCs [439,440]. A self-inactivating lentiviral vector carrying a functional *RPS19* transgene is currently under clinical evaluation for *RPS19*-deficient DBAS (NCT07476183).

In malignancy, hyperactive RiBi represents a therapeutic vulnerability. Future DNA-based strategies may offer opportunities to restore dysregulated RiBi or selectively disrupt nucleolus-dependent proliferation. Candidate targets could include NPM1, RPs, and *MYC*, which are frequently overexpressed in cancers [63,240,241,242,249]. Approaches such as non-integrating vectors or *in vivo* gene editing could also provide opportunities for direct tumour targeting with reduced off-target risk.

#### 7.3.2. RNA-Based Therapeutic Strategies

RNA-based therapies enable transient and tuneable modulation of protein expression and have been applied across diverse therapeutic modalities, including protein replacement, vaccines, immunotherapy, and gene editing, with applications across a wide range of diseases such as cancer, infectious, immune, haematological, metabolic, cardiovascular, respiratory, and neurological disorders [441,442,443,444]. RNA-based approaches are particularly useful when permanent genomic modification is undesirable or when delivery constraints limit DNA-based strategies. In the context of nucleolar disorders, RNA therapeutics could be employed to correct RiBi defects in ribosomopathies or selectively induce nucleolar stress in cancer cells.

##### mRNA-Based Protein Replacement and Gene Editing

Synthetic mRNA may replace defective RPs or nucleolar regulators, offering a temporary compensatory mechanism for mutations associated with ribosomopathies. Lipid nanoparticles (LNPs) enable efficient HSPC uptake *in vivo* and transient protein expression without genome integration [445,446,447,448,449,450,451]. mRNA approaches have corrected disease phenotypes in preclinical models of erythroid disorders, including haemophilia A [452] and B [453], thrombotic thrombocytopenic purpura [454], β-thalassemia [449], sickle cell disease [445], and Fanconi anaemia [448]. Although untested in ribosomopathies, this approach may provide a strategy to support ribosome assembly in HSPCs. Beyond protein replacement, mRNA can encode gene-editing machinery, enabling precise correction of pathogenic alleles [455,456]. mRNA-based platforms may also enable expression of non-genotoxic conditioning factors for HSCT; for example, by using transient expression of pro-apoptotic factors to selectively deplete HSPCs, reducing reliance on chemotherapy or irradiation [445].

In the context of cancer, mRNA-based strategies could restore tumour suppressor function or sensitise tumour cells to adjunct therapies [457]. In preclinical models, delivery of *TP53* mRNA restored p53 protein expression in *TP53*-null non-small-cell lung cancer (NSCLC) and hepatocellular carcinoma (HCC), suppressing tumour growth and increasing sensitivity to the mTOR inhibitor everolimus [458]. *Trp53* mRNA has also been shown to enhance the efficacy of anti-PD-1 immunotherapy in HCC models by reshaping the immune tumour microenvironment, resulting in reduced tumour growth and improved survival [459]. Similarly, *PTEN* mRNA delivery restored functional PTEN and reversed the immunosuppressive tumour microenvironment in PTEN-deficient prostate tumours and melanoma [460], and lysine-specific demethylase 6A (*KDM6A*) mRNA inhibited metastasis in *KDM6A*-null bladder cancer models [461]. mRNA delivery could also be used to induce nucleolar stress in tumour cells, providing a potential strategy to exploit nucleolar dependencies in cancer.

##### RNA Editing

RNA-editing technologies, including RNA trans-splicing and base editing, may enable correction of mutant transcripts encoding nucleolar proteins without altering genomic DNA [462,463]. In ribosomopathies, such approaches could recover functional RP expression, while in cancer, they may enable correction of nucleolar driver mutations. Adenine base editors have been used to repair *TP53* hotspot mutations in cancer cell lines from multiple tissues, restoring p53 tumour-suppressive programs and inhibiting cancer cell growth [464]. This platform has also corrected cancer-associated mutations in *KRAS*, *PTEN*, and *SMAD4* [464]. Although largely proof-of-concept, even partial transcript correction could provide therapeutic benefit and act synergistically with chemotherapy [465]. Key challenges include delivery efficiency, off-target activity, and mutation-specific design, although advances in RNA delivery and editing are progressively addressing these issues [463,466].

##### ASO and siRNA-Based Therapeutic Strategies

Antisense oligonucleotides (ASOs) and siRNAs may provide transcript-level modulation of nucleolar proteins [467]. In ribosomopathies, ASOs could potentially correct aberrant splicing or reduce dominant-negative transcripts, although preclinical studies remain limited [468]. In nucleolus-driven cancers, future therapeutic strategies may employ siRNA-mediated knockdown of overexpressed nucleolar regulators, such as NPM1, RPs, and c-Myc, to induce nucleolar stress and impair tumour proliferation, with preclinical studies demonstrating efficacy in reducing oncogene expression [469,470,471,472,473,474,475,476,477,478,479,480]. However, no cancer siRNA therapies targeting nucleolar pathways have yet reached clinical approval.

Advantages of these approaches include high specificity, transient and tuneable effects, and compatibility with delivery platforms such as LNPs or GalNAc conjugates [467,474,480,481]. Limitations include efficient delivery to HSPCs or solid tumours, RNA stability, immunogenicity, and lack of clinical validation [467,474,480,481]. Future strategies may integrate ASO or siRNA therapies with mRNA replacement or DNA-based editing to simultaneously suppress pathogenic transcripts and restore protein function.

#### 7.3.3. Delivery Challenges

Targeted delivery to the desired tissue and cell type remains a central challenge for both DNA- and RNA-based therapies [482,483]. In particular, HSPCs are difficult to access due to their low abundance, protective niches, and sensitivity to manipulation, while selective targeting of tumour cells requires high precision [482,483,484]. Recent advances are nevertheless improving targeted delivery to these cell types; LNPs engineered with cell-specific ligands can achieve *in vivo* transfection without *ex vivo* manipulation. For example, modified *Fancc* mRNA delivered via LNPs restored HSPC expansion in *Fancc*^–/–^ mice [448]. Similarly, CD117- or CD45-targeted antibody-conjugated and antibody-free LNPs have achieved HSPC transfection in preclinical models, highlighting the potential for non-genotoxic *in vivo* delivery [445,446,447,448,449,450,451].

DNA- and RNA-based therapies provide a promising route to address ribosomal dysfunction in ribosomopathies and exploit nucleolus-dependent vulnerabilities in cancer, with early preclinical studies supporting their potential to restore RP expression and modulate nucleolar stress pathways. However, translating this potential will require advances in targeted delivery, expression durability, immune tolerance, and scalable production [442,463,467,483]. Addressing these constraints will be critical for realising the broader clinical potential of DNA- and RNA-based therapeutic platforms, including their application to disorders of nucleolar function and cancers in which the nucleolus represents a tractable therapeutic target.

## 8. Conclusions and Future Perspectives

The nucleolus has evolved from being viewed as a specialised site of ribosome production to a central regulator of cellular homeostasis. Across the areas covered in this review, a consistent theme emerges: RiBi is tightly integrated with growth and stress signalling, and disruption of this coupling has direct consequences for cell fate. The nucleolus functions accordingly as a sensor and integrator of cellular conditions, linking rDNA transcription to nucleolar surveillance pathways and broader stress-response networks.

This provides a unifying model for its role in disease. In cancer, elevated RiBi supports proliferative and metabolic demand; in ribosomopathies, impaired ribosome production engages stress pathways that constrain cell growth or survival. These opposing phenotypes reflect a shared underlying principle: cellular fitness is acutely sensitive to changes in ribosome output, with the NSP acting as a central mediator. The nucleolus therefore sits upstream of many of the pathological phenotypes described, rather than arising as a secondary consequence of them.

Several important questions remain unresolved, including how nucleolar organisation and function are remodelled across cellular states, how distinct nucleolar outputs are specified in a context-dependent manner, and the extent to which p53-independent pathways contribute to pathology. Resolving these questions will be essential for translating mechanistic insight into therapeutic progress.

The therapeutic landscape is advancing. Current approaches largely suppress elevated RiBi in cancer or compensate for downstream defects in ribosomopathies, but emerging strategies indicate that more direct and selective modulation of nucleolar function is achievable. Small-molecule inhibitors of Pol I transcription, gene-editing strategies, and RNA-based approaches offer complementary routes to manipulate nucleolar pathways, and advances in delivery are making cell-type-specific intervention increasingly feasible. Improved methods to resolve nucleolar composition and function are concurrently revealing tractable therapeutic vulnerabilities.

The nucleolus is not a passive downstream effector of disease but an underexploited point of control. The next step is to move beyond broad suppression or compensation towards mechanism-informed, context-specific modulation of nucleolar activity, and the tools to do so are within reach. As mechanistic understanding improves, interventions that act directly on RiBi and nucleolar surveillance pathways offer a credible and increasingly realistic path to greater therapeutic specificity and durability across a range of disease settings.

## Data Availability

No new data were created or analysed in this study. Data sharing is not applicable to this article.
